# Social and non-social working memory in neurodegeneration

**DOI:** 10.1016/j.nbd.2023.106171

**Published:** 2023-05-29

**Authors:** Agustina Legaz, Pavel Prado, Sebastián Moguilner, Sandra Báez, Hernando Santamaría-García, Agustina Birba, Pablo Barttfeld, Adolfo M. García, Sol Fittipaldi, Agustín Ibañez

**Affiliations:** a Cognitive Neuroscience Center (CNC), Universidad de San Andres, Buenos Aires, Argentina; b National Scientific and Technical Research Council (CONICET), Buenos Aires, Argentina; c Universidad Nacional de Córdoba, Facultad de Psicología, Córdoba, Argentina; d Latin American Brain Health Institute (BrainLat), Universidad Adolfo Ibañez, Santiago, Chile; e Global Brain Health Institute (GBHI), University of California San Francisco (UCSF), San Francisco, United States; f Escuela de Fonoaudiología, Facultad de Odontología y Ciencias de la Rehabilitacion, Universidad San Sebastián, Santiago, Chile; g Universidad de los Andes, Bogotá, Colombia; h Pontificia Universidad Javeriana, Medical School, Physiology and Psychiatry Departments, Memory and Cognition Center Intellectus, Hospital Universitario San Ignacio, Bogotá, Colombia; i Cognitive Science Group. Instituto de Investigaciones Psicológicas (IIPsi), CONICET UNC, Facultad de Psicología, Universidad Nacional de Córdoba, Boulevard de la Reforma esquina Enfermera Gordillo, CP 5000. Córdoba, Argentina; j Facultad de Psicología, Universidad de La Laguna, Tenerife, Spain; k Instituto Universitario de Neurociencia, Universidad de La Laguna, Tenerife, Spain; l Departamento de Lingüística y Literatura, Facultad de Humanidades, Universidad de Santiago de Chile, Santiago, Chile; m Trinity College Dublin (TCD), Dublin, Ireland

**Keywords:** Working memory, social processing, social working memory, behavioral-variant frontotemporal dementia, Alzheimer’s disease, Parkinson’s disease

## Abstract

Although social functioning relies on working memory, whether a social-specific mechanism exists remains unclear. This undermines the characterization of neurodegenerative conditions with both working memory and social deficits. We assessed working memory domain-specificity across behavioral, electrophysiological, and neuroimaging dimensions in 245 participants. A novel working memory task involving social and non-social stimuli with three load levels was assessed across controls and different neurodegenerative conditions with recognized impairments in: working memory and social cognition (behavioral-variant frontotemporal dementia); general cognition (Alzheimer’s disease); and unspecific patterns (Parkinson’s disease). We also examined resting-state theta oscillations and functional connectivity correlates of working memory domain-specificity. Results in controls and all groups together evidenced increased working memory demands for social stimuli associated with frontocinguloparietal theta oscillations and salience network connectivity. Canonical frontal theta oscillations and executive-default mode network anticorrelation indexed non-social stimuli. Behavioral-variant frontotemporal dementia presented generalized working memory deficits related to posterior theta oscillations, with social stimuli linked to salience network connectivity. In Alzheimer’s disease, generalized working memory impairments were related to temporoparietal theta oscillations, with non-social stimuli linked to the executive network. Parkinson’s disease showed spared working memory performance and canonical brain correlates. Findings support a social-specific working memory and related disease-selective pathophysiological mechanisms.

## 1. Introduction

Working memory (WM) plays a critical role in cognition and social functioning by allowing the maintenance and manipulation of information (Christophel et al., 2017; Porcelli et al., 2019) within load-dependent limits (Cowan, 2017; Oberauer et al., 2016). Yet, it is still unclear whether social stimuli involve domain-general or -specific WM processes. Although most traditional studies have targeted non-social stimuli (Chai et al., 2018; Meyer and Lieberman, 2012), relevant works suggest social stimuli increase WM load resembling a ‘social impairment’ effect (i.e., reduced WM performance linked to social processing in comparison to non-social cues) (Fairfield et al., 2015; Garrison and Schmeichel, 2019; Pessoa, 2009; Plancher et al., 2019). However, the field is not without controversy. Social stimuli maintenance and manipulation have been linked to canonical non-social WM hubs -i.e., the frontoparietal executive network (EN) (Smith et al., 2017; Thornton and Conway, 2013; Xin and Lei, 2015) suggesting a domain-general WM for social stimuli. Few works have related WM for social stimuli to a broader network beyond executive regions -i.e., social processing hubs including medial frontal, cingulate, and temporoparietal areas (Meyer and Collier, 2020; Meyer et al., 2012; Meyer et al., 2015), proposing a domain-specific WM subsystem [or social WM (Meyer and Lieberman, 2012)]. This scenario limits the characterization of clinical conditions with deficits in both WM and social processes. Neurodegenerative lesion models can partially overcome correlational evidence by exposing direct associations between behavioral performance and critical brain regions (Birba et al., 2022; Cruzat et al., 2023; Legaz et al., 2022; Moguilner et al., 2022; Rorden and Karnath, 2004; Salamone et al., 2021; Santamaría-García et al., 2022). To our knowledge, no previous work has targeted the WM domain-specificity for social vs non-social stimuli across neurodegenerative conditions with different WM and socio-cognitive impairments, such as behavioral-variant frontotemporal dementia (bvFTD), Alzheimer’s disease (AD), and Parkinson’s disease (PD) (Piguet et al., 2011; Salmi et al., 2020) –let alone with a multidimensional approach indexing behavioral, electrophysiological (resting-state electroencephalography [rsEEG]) and neuroimaging (resting-state functional magnetic resonance imaging [rsfMRI]) methods. Answering these questions is relevant for both cognitive and translational neuroscience.

The bvFTD presents non-social WM alterations (Poos et al., 2018) predominantly related to frontal (Nissim et al., 2017) and secondarily to temporoinsular atrophy (Baez et al., 2019; Migeot et al., 2022; Possin et al., 2013). However, WM dynamics for social stimuli remain unexamined in bvFTD. Electrophysiological WM correlates also remain unassessed, beyond general frontotemporal theta (θ) oscillatory disruption (Caso et al., 2012; Metin et al., 2018). Social processing deficits are pervasive in bvFTD (Dodich et al., 2021; Ibáñez and Manes, 2012; Kipps et al., 2009; O’Callaghan et al., 2016; Piguet et al., 2011) and have been linked to frontoinsular and temporoparietal atrophy (Baez et al., 2019; Kumfor et al., 2017), reduced fronto-posterior electrophysiological activity (Melloni et al., 2016), and salience network (SN) dysfunctions (Rijpma et al., 2022; Toller et al., 2018). Nonetheless, it is unknown whether a domain-general or -specific WM disruption exists in this condition with both WM and social processing deficits.

AD presents non-social WM impairments (Kirova et al., 2015) comparable to bvFTD (Leslie et al., 2016; Ramanan et al., 2017a), although linked to left temporoparietal dysfunctions (Kobylecki et al., 2018) and to reduced frontoparietal θ oscillations (Goodman et al., 2019; Hata et al., 2016). Indeed, altered WM in AD may be explained by global cognitive deficits (Possin et al., 2013) and related to EN dysfunctions (Agosta et al., 2012; Ibáñez et al., 2021a; Moguilner et al., 2021; Tait et al., 2020; Zhao et al., 2019). Yet, how social stimuli modulate WM in AD has not been assessed. Particularly, social processing impairments related to default mode network (DMN) dysfunctions (Badhwar et al., 2017; Saris et al., 2021) have also been linked to AD severity and global cognitive deficits (Dodich et al., 2016; Musa et al., 2020; Ramanan et al., 2017b; Synn et al., 2018). In sum, the extent of social specificity over an altered WM remains unexamined in this general cognitive deficit model.

Finally, although non-social WM deficits have been reported in PD (Ramos and Machado, 2021), dopaminergic-medicated PD patients tend to perform as well as healthy controls (HCs) (Moustafa et al., 2013; Salmi et al., 2020) with increased compensatory frontal activity despite corticostriatal disruptions (Simioni et al., 2017). In fact, the EN is spared in this disease (Hou et al., 2018). Again, no previous work has studied how social stimuli modulate WM in PD. Indeed, social processing disturbances are still inconclusive (Argaud et al., 2018; Lewis and Ricciardi, 2021; Maresca et al., 2020). Altogether, it is not clear whether WM is selectively impaired for social stimuli in this unspecified disease model.

Briefly, the notion of distinct WM mechanisms for social relative to non-social stimuli remains untested in bvFTD, AD, and PD -let alone combining behavioral and neurofunctional dimensions. To fill this gap, we employed a novel domain-specific WM task with social and non-social stimuli across these conditions. The task required participants to identify the sameness between separately presented lists of social (e.g., *cordial*) and non-social (e.g., *oval*) adjectives, organized in three load levels: three (triplets), four (quartets), or five (quintets) words. In addition, we acquired offline rsEEG modulations to capture θ oscillations (~4–8 Hz) given their systematic association to verbal WM load (Dai et al., 2017; Pavlov and Kotchoubey, 2020) and social processing (Billeke et al., 2013; Gregory et al., 2021). Finally, offline rsfMRI was also obtained to investigate functional connectivity correlates of WM domain-specificity [three networks linked to WM and social processes: the SN, the EN, and the DMN (Feng et al., 2021); and two control networks: the visual network (VN), and the motor network (MN)].

We propose distinct hypotheses for each group. In HCs, we predicted a behavioral WM load effect (triplets > quartets > quintets), with lower performance for social vs non-social stimuli (Fairfield et al., 2015; Garrison and Schmeichel, 2019). Increased WM for social stimuli should be linked to higher θ oscillations in extended frontocinguloparietal regions [related to social processing (Billeke et al., 2013; Feng et al., 2021; Gregory et al., 2021) and WM (Constantinidis and Klingberg, 2016; Dai et al., 2020)]; as well as higher SN connectivity (Luo et al., 2014; Rijpma et al., 2021). Conversely, WM for non-social stimuli should be associated with θ oscillations in canonical right frontal regions (Dai et al., 2017; Pavlov and Kotchoubey, 2020) as well as higher EN and lower DMN connectivity (Chai et al., 2018; Liang et al., 2016; Maehara, 2017). A WM load-dependent enhancement of θ oscillations should be observed irrespective of stimulus type (Dai et al., 2017; Pavlov and Kotchoubey, 2020). Compared to HCs, each patient group should present distinct WM patterns. In bvFTD, we expected increased WM deficits for social vs non-social stimuli, linked to θ oscillations in fronto-posterior hubs, and to SN connectivity. In AD, we hypothesized WM deficits in both social and non-social stimuli, predominantly associated to temporoparietal θ oscillations and EN connectivity. In PD, we predicted preserved WM, associated with frontal θ oscillations. A behavioral WM load effect was also expected in all neurodegenerative groups. By testing these hypotheses, we aim to provide multimodal evidence of social specificity in WM across neurodegenerative models.

## 2. Methods

### 2.1. Participants

**Fig. 1.**
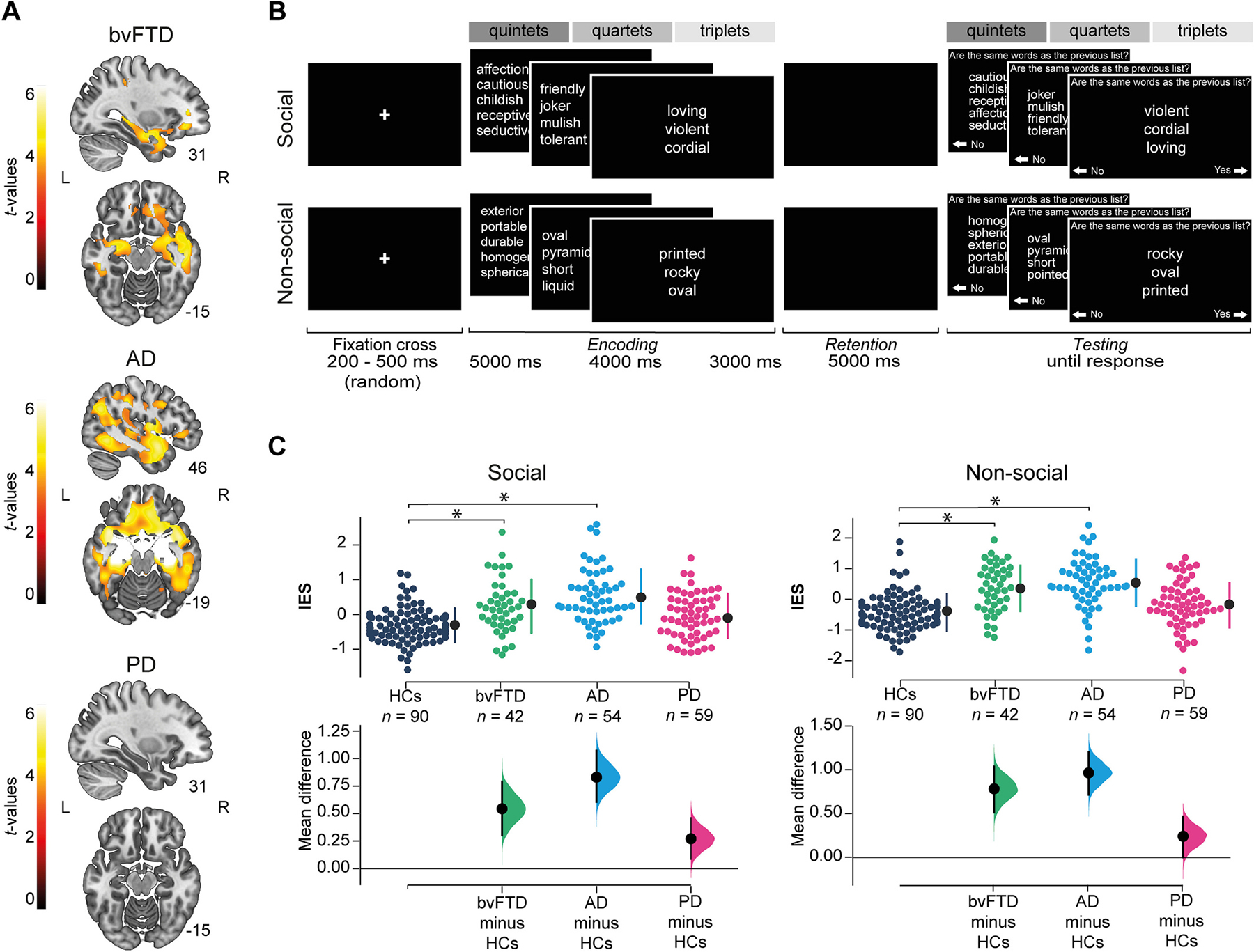
GM atrophy, task design and behavioral results. (A)GM atrophy in patients. GM integrity was assessed via voxel-based morphometry, based on *w*-score maps of the normalized and smoothed DARTEL outputs (Chung et al., 2017; Jack et al., 1997; La Joie et al., 2012; Ossenkoppele et al., 2015; van Loenhoud et al., 2017). We ran two sample *t*-tests between each neurodegenerative group and HCs using the statistical non-parametric mapping (SnPM13) toolbox for SPM12 (5000 random permutations, *P*<0.001 for cluster-forming threshold, and *P*<0.05 FWE-corrected for cluster-wise threshold (Kim et al., 2020; Salamone et al., 2021; Shih et al., 2019)). BvFTD showed orbitofrontal-cingulate-temporal atrophy. AD showed bilateral temporal with less extended frontoparietal atrophy. No atrophy was found in PD (Table S1.2). Results are presented on MNI space using the AAL(Tzourio-Mazoyer et al., 2002), in neurological convention. (B) Task design. Participants judged if adjectives from a second list (*testing* phase) were the same as those from a first list (*encoding* phase) after a *retention* phase. Adjectives were either social or non-social (stimulus type) and randomly presented in three load levels: quintets, quartets or triplets (dark, medium and light gray, respectively). Note: Adjectives were displayed and validated in Spanish (Material S2). English translations are simply communicative renditions for the benefit of non-Spanish readers. (C)Behavioral results: between-group comparisons. We compared the WM performance of HCs and patient groups via mixed model ANOVA (group[4]*type[2]*load[3]) and post-hoc Tukey comparisons using the normalized inverse efficiency score (IES). Significant results were found for group-by-type (plotted) and group-by-load interactions (Table 2, Fig. S1, Fig. S2, Material S4.) Dot-plots represent results for HCs (dark blue), bvFTD (turquoise), AD (light blue), and PD (pink). Vertical-dotted lines show mean (black dot) and standard deviation (lines). The asterisk indicates significant differences (*P*<0.05). The between-groups mean difference (effect size) between each patient group and HCs is reported below each result. AD: Alzheimer’s disease, bvFTD: behavioral-variant frontotemporal dementia, GM: grey matter, HCs: healthy controls, L: left, PD: Parkinson’s disease, R: right, WM: working memory.

The study was coordinated by BrainLat (Duran-Aniotz et al., 2022) and comprised 245 participants: 90 HCs with preserved cognition and no history of neuropsychiatric diseases and/or substance abuse; 42 people fulfilling revised criteria for bvFTD (Rascovsky et al., 2011); 54 people with AD, each meeting the international NINCDS-ADRDA criteria (Dubois et al., 2007; McKhann et al., 2011); and 59 people with PD diagnosed in accordance with the United Kingdom PD Society Brain Bank criteria (Hughes et al., 1992). Power analyses confirmed the adequacy of our sample size (Material S1.1). Participants were recruited from four international clinics taking part in the Multi-Partner Consortium to Expand Dementia Research in Latin America (ReDLat) and assessed following harmonized procedures (Ibáñez et al., 2021b; Ibáñez et al., 2021c; Maito et al., 2023; Moguilner et al., 2023) as in previous reports (Donnelly-Kehoe et al., 2019; Ibáñez et al., 2021a; Legaz et al., 2022; Melloni et al., 2016; Salamone et al., 2021; Sedeno et al., 2017). Clinical diagnoses were established by experts through an extensive neurological, neuropsychiatric, and neuropsychological examination comprising semi-structured interviews and standardized cognitive assessments (Table 1). Participants with neurodegenerative conditions were in early/mild stages of the disease. They did not fulfill criteria for other neurological, psychiatric and/or primary language disorders, or a history of substance abuse. As verified by caregivers, bvFTD and AD participants were functionally impaired, with bvFTD exhibiting prominent changes in personality and social behavior. PD participants were medicated with antiparkinsonian therapy (dopaminergic medication) and evaluated during the ‘ON’ phase. Each neurodegenerative sample was comparable in sex, age, and years of formal education with HCs (Table 1). Finally, whole-brain GM was compared between each neurodegenerative group and HCs, showing a predominantly orbitofrontal-cingulate-temporal atrophy in bvFTD (Ibáñez and Manes, 2012; Whitwell et al., 2009), bilateral temporal with less extended frontoparietal atrophy in AD (Du et al., 2007; Landin-Romero et al., 2017; Pini et al., 2016), and no atrophy in PD (Huber et al., 1989; Price et al., 2004; Schulz et al., 1999) (Fig. 1A; Table S1.2). The institutional ethics committee of each recruitment center approved the study protocol. All participants provided signed informed consent in accordance with the Declaration of Helsinki.

### 2.2. Experimental protocol

Participants completed a multimodal assessment protocol including a behavioral WM task, and offline resting-state high-density EEG recordings and MRI-fMRI sessions.

#### 2.2.1. Behavioral data: domain-specific WM task

Behavioral data was obtained through a domain-specific WM task (Fig. 1B). It consists of the sequential presentation of two lists of words. Participants are asked to judge whether the words in the second list are all the same as those from the first list (beyond word’s order) by pressing predefined keys. The task comprises two stimulus types: social, where words are adjectives that describe the interaction between two persons (e.g., *cordial, friendly, prudent*); and non-social, where words are adjectives that cannot be used to describe a person during a social interaction (e.g., *oval, rocky, printed*). Moreover, the task includes three load levels: social and non-social adjectives are organized in lists of three (triplets), four (quartets) and five (quintets) words. We manipulated the content of the stimuli (social/non-social) in order to compare their effect on WM, and the load (triplets, quartets, quintets) to confirm task understanding and control for potential cognitive confounds. The task was designed based on previous WM paradigms (Fiebach et al., 2006; Parra et al., 2010; Pietto et al., 2016; Reuter-Lorenz et al., 2000). Social and non-social adjectives were validated through online surveys distributed across Spanish speaking countries. We controlled that words between social and non-social trials were statistically paired for lexical parameters (log frequency, number of letters, number of syllables) and for Levenshtein distance. Conversely, they were statistically different for sociability. We also controlled that triplets, quartets and quintets were statistically paired for lexical parameters within stimulus type. Full details of task design and validation are provided in Material S2.

Each trial consists of three phases: *(a) encoding, (b) retention* and *(c) testing*. They initiate with a fixation cross (random duration between 200 – 500 ms). *(a)* Then stimuli are presented (first list of triplets, quartets, or quintets). The duration depends on the stimuli load: 3000 ms for triplets, 4000 ms for quartets, and 5000 ms for quintets (Morrison et al., 2016). *(b)* Immediately after, a black screen appears for 5000 ms. *(c)* Finally, a second list of words (same load as the first list) is shown until the subject’s response, with ‘No’/’Yes’ options positioned to the bottom left and right of the screen next to left/right arrows, respectively. Participants have to respond the sameness of the words lists by choosing ‘No’/’Yes’ through the corresponding computer keyboard arrows with their dominant hand. In the *testing* phase, half of the lists are the same as in the *encoding* phase, and half are different. For the same lists, only the word’s order is changed. For the different lists, one word is replaced by another with the same initial syllable/letter (similarity between old and new words did not differ between social and non-social conditions, as assessed by Levenshtein distance [minimum number of changes required to convert one word into the other] – see Table S2.1.3). Instructions and a set of six practice trials are presented before the task. The order of trials per stimulus type, load level, and equality between first and second list is randomly assigned. In total, participants complete 60 trials: 20 trials per load (10 social, 10 non-social). The number of trials does not change according to performance. Approximately, the total duration of the task is 15 minutes. Accuracy and response time (RT) data were collected for each trial.

#### 2.2.2. EEG: acquisition and signal preprocessing

We acquired 10-minute, high-density offline rsEEG (eye-closed) recordings from a 91-participants subsample. Signals were recorded using a Biosemi ActiveTwo 128-channel acquisition system with pre-amplified sensors and a DC coupling amplifier. Reference electrodes were set to linked mastoids. Analog filters were set at 0.03 and 100 Hz. Signals were sampled at 1024 Hz. The subsample comprised 19 bvFTD, 27 AD, and 12 PD, each group being demographically matched with HCs (*n* = 33) (see Table S3.1). Participants were instructed to remain still and awake, while sitting in a comfortable chair, inside a dimly lit sound-attenuated and electromagnetically-shielded EEG chamber.

rsEEG signals were preprocessed offline using standard procedures in MATLAB’s EEGLAB toolbox (Delorme and Makeig, 2004). Recordings were band-pass filtered at 0.5–40 Hz, and re-referenced to the average of all channels. Malfunctioning channels were identified and replaced using statistically weighted spherical interpolation (based on neighbor sensors) (Courellis et al., 2016). Data was down-sampled to 512 Hz. Eye movements or blink artifacts were corrected with independent component analysis (Kim and Kim, 2012) and with a visual inspection protocol (Birba et al., 2021; Dirlich et al., 1997; Garcia-Cordero et al., 2017; Garcia-Cordero et al., 2016; Pollatos and Schandry, 2004; Salamone et al., 2021; Schandry and Montoya, 1996).

#### 2.2.3. Neuroimaging: acquisition and preprocessing

MRI and rsfMRI acquisition and preprocessing steps are reported as recommended by the Organization for Human Brain Mapping (Nichols et al., 2017; Poldrack et al., 2017). Following standard protocols (Garcia-Cordero et al., 2016; Gonzalez Campo et al., 2019), we obtained offline three-dimensional volumetric and 10-minute rsfMRI sequences from a subsample of 165 participants. These comprised 19 bvFTD, 32 AD, and 48 PD, each group being demographically matched with HCs (*n* = 66) (see Table S3.2). Recordings were performed in different scanners (for harmonization details see Table S3.3). Participants were asked not to think about anything in particular, move or fall asleep. To avoid noisy signals coming from the visual cortex, we chose the closed-eyes modality (Zou et al., 2015).

First, to ensure that magnetization achieved a steady state, we discarded the first five volumes of each subject’s resting-state recording. Then, images were preprocessed using the Data Processing Assistant for Resting-State fMRI (DPARSF V2.3) (Chao-Gan and Yu-Feng, 2010) open-access toolbox in MATLAB, which generates an automatic pipeline for fMRI analysis by calling the Statistical Parametric Mapping software (SPM12) and the Resting-State fMRI Data Analysis Toolkit (REST V.1.7). As in previous studies (Salamone et al., 2021; Yoris et al., 2018), preprocessing steps included slice-timing correction (using middle slice of each volume as the reference scan) and realignment to the first scan of the session to correct head movement (SPM functions) (Barttfeld et al., 2012; Garcia-Cordero et al., 2016; Melloni et al., 2016; Sedeno et al., 2016). Then, images were normalized to the MNI space using the default echo-planar imaging template from SPM12 (Ashburner and Friston, 1999), smoothed using an 8-mm full-width-at-half-maximum isotropic Gaussian kernel, and bandpass filtered between 0.01–0.08 Hz to correct and remove low-frequency drifts from the scanner. Finally, we regressed out six motion parameters, cerebrospinal fluid, and white matter signals to reduce motion and physiological artifacts such as cardiac and respiration effects (REST V1.7). Motion parameters were estimated during realignment, and cerebrospinal fluid and white matter masks were derived from the tissue segmentation of each subject’s T1 scan in native space with SPM12 (after co-registration of each subject’s structural image with the functional image). Finally, we excluded recordings with movements greater than 3 mm and/or rotation higher than 3° (Table S3.4) (Supekar and Menon, 2012; Supekar et al., 2008).

### 2.3. Statistical analysis

#### 2.3.1. Behavioral analysis: domain-specific WM task

First, to improve the responses’ signal-to-noise ratio and avoid random responses, we removed trials with RTs < 250 ms and then excluded those trials falling three Median Absolute Deviations (MADs) away from the median of each subjects’ RT. This approach has proven to be more robust against outliers’ effect compared to SDs from the mean (Dave and Varma, 2014; Yang et al., 2019). Next, to ensure non-significant differences in the final number of trials between conditions, we randomly selected and kept the same number of trials per stimulus type and load level, for each subject (for data curation details see Fig. S1, Table S4.1, and Table S4.2). Finally, to quantify WM performance, we used the inverse efficiency score (IES) (Hesse et al., 2016; Hesse et al., 2019), a standard metric that combines accuracy and RT to holistically establish weighted behavioral outcomes (Jacques and Rossion, 2007; Jacquet and Avenanti, 2015), previously applied in neurodegenerative studies (Salamone et al., 2021). The IES is calculated by dividing the mean RT by the proportion of correct responses (Brozzoli et al., 2008; Mevorach et al., 2006), thus controlling for biases introduced by fast RTs with low accuracy and vice versa. Therefore, the higher the IES, the poorer the performance. Average IES scores were calculated for each stimulus type (social, non-social) and load level (triplets, quartets, quintets), per subject. Given that Shapiro-Wilk’s tests revealed a non-normal distributions for IES indexes, and that analyses based on non-normalized data may promote Type I and Type II errors (Rasmussen, 1985), IES scores were normalized using Ordered Quantile Normalization transformation (Table S4.3, Table S4.4), previously applied in literature (Das et al., 2022; Minnier et al., 2021). Based on a rank mapping of the observed data to the normal distribution, this technique guarantees normally distributed transformed data if ties are not present (Peterson and Cavanaugh, 2019).

Second, we run mixed ANOVA models of the IES across stimulus type and load level (2 [type] * 3 [load]) for each group separately. These analyses confirmed the task validation regarding the expected ‘social impairment’ effect in HCs and the load effect at each group (for further details see Table S4.5 and Fig. S2).

Finally, since our main hypotheses hinged on differences between each patient group and HCs, statistical analyses of behavioral data were performed to compare group pairs of patients and controls (bvFTD vs HCs, AD vs HCs, PD vs HCs) as in previous reports with neurodegenerative conditions (Chiong et al., 2016; Garcia-Cordero et al., 2016; Garcia-Cordero et al., 2019; Legaz et al., 2022; Salamone et al., 2021; Shany-Ur et al., 2014; Shany-Ur et al., 2012; Sollberger et al., 2014; Sollberger et al., 2009). IES was first compared between groups via mixed model ANOVAs, for each stimulus type and load level (4 [group] * 2 [type] * 3 [load]). Then, specific WM patterns in each patient group relative to HCs were assessed through pairwise comparisons (bvFTD-HCs, AD-HCs, and PD-HCs) via Tukey’s HSD tests. This procedure accounts for multiple comparisons reducing the probability of Type I error (Nanda et al., 2021). Effect sizes were reported with partial eta squared (*ηp*^*2*^). Behavioral analyses were performed using BestNormalize (Peterson and Cavanaugh, 2019), lmerTest (Kuznetsova et al., 2017), afex (Singmann et al., 2015), and effectsize (Ben-Shachar et al., 2020) packages in R software (Version 4.0.2, R Foundation for Statistical Computing). Figures were generated using the Seaborn Python package (Version 0.9.0) (Waskom, 2021).

#### 2.3.2. EEG: source localization analysis

After preprocessing, we conducted a source analysis of the rsEEG in the frequency domain using the standardized Low-Resolution Electromagnetic Tomography method [sLORETA (Grech et al., 2008; Pascual-Marqui, 2002)] to examine associations between behavioral WM outcomes and θ oscillations correlates related to WM (Dai et al., 2017; Pavlov and Kotchoubey, 2020) and social processing (Billeke et al., 2013; Gregory et al., 2021). First, we computed the EEG cross-spectrum at the sensor level from the discrete Fourier transforms obtained for each EEG channel, using a 2s-length window. Then, the cross-spectrum was used to calculate the standardized current density maps. These maps were obtained using a three-concentric-spheres head model, in a predefined source space of 6239 voxels (voxel size of 5 × 5 × 5 mm^3^) of the MNI average brain (Evans et al., 1993). A brain segmentation of 82 anatomic compartments (cortical areas) was implemented using the automated anatomical labeling (AAL) atlas (Tzourio-Mazoyer et al., 2002). Current densities maps of each participant were frequency-wise normalized. For each frequency, the spectral power computed in each voxel was divided by the mean spectral power (6239 voxels’ average) (for further details see Material S5.1).

Normalized current densities maps computed in the θ frequency band of the EEG were correlated with behavioral outcomes (IES) for both, stimulus type (social and non-social) and the most extreme load level (quintets [high] and triplets [low]) conditions, using Pearson correlation tests. To adjust for multiple comparisons and reduce the probability of Type I error, we used false discovery rate (FDR) rate (*P* < 0.05 FDR-corrected) (Benjamini and Yekutieli, 2001; Bennett et al., 2009; Brancaccio et al., 2020). To increase behavioral variance and statistical power, analyses collapsing all groups together (HCs, bvFTD, AD, and PD) were added to the individual group’s correlation analyses (Garcia-Cordero et al., 2016; O’Callaghan et al., 2016; Sollberger et al., 2009).

#### 2.3.3. Neuroimaging: resting-state functional connectivity analysis

After preprocessing, we used seed analyses to examine associations between behavioral WM outcomes and dynamic functional connectivity across three core brain networks associated to social processing and WM (Feng et al., 2021): the SN, related to salient-social information processing (Porcelli et al., 2019; Toller et al., 2018; Uddin, 2015); the EN, implicated in externally goal-oriented executive processes including WM (Constantinidis and Klingberg, 2016; Menon and D’Esposito, 2022); and the DMN, that supports internally-related processes (Smallwood et al., 2021) and inversely correlates with the EN activation during high load WM processes (Liang et al., 2016). To test the specificity of our predictions for these networks, we also examined associations between WM and connectivity along two additional unrelated networks: the VN and the MN. To calculate each resting-state network connectivity, we located bilateral seeds on different MNI coordinates for each network (Koslov et al., 2011) (for further details see Material S5.2). Then we employed a weighted Symbolic Dependence Metric (wSDM) non-linear correlation coefficient across the whole time series obtained in the resting-state acquisition which proved robust in neurodegenerative conditions (Moguilner et al., 2018). After that, we used standard masks (Shirer et al., 2012) to isolate the voxels that are typically involved in each resting-state network. Finally, we spatially averaged across all included voxels to obtain one feature per network. Resulting connectivity maps were correlated with behavioral outcomes (IES) for both, stimulus type (social and non-social) and the most extreme load level (quintets [high] and triplets [low]) conditions, through the SPM12 multiple regression module. To adjust for multiple comparisons, we used cluster-wise inference with false discovery rate (FDR) rate correction (*P*≤0.05 FDR-corrected) (Han et al., 2019). In line with the EEG analysis pipeline, in addition to individual groups correlation analyses, we performed analyses collapsing all groups together (HCs, bvFTD, AD, and PD) to increase behavioral variance and statistical power (Garcia-Cordero et al., 2016; O’Callaghan et al., 2016; Sollberger et al., 2009).

### 2.4. Availability of data and materials

The datasets supporting the conclusions of this article are publicly available in the OSF repository, http://osf.io/bx27h (Legaz, 2022).

## 3. Results

### 3.1. Behavioral results

Our analysis revealed significant main effects for group (*F*_3,240_ = 25.22, *P* < 0.001, *ηp*^*2*^ = 0.24) and load (*F*_2,480_ = 408.61, *P* < 0.001, *ηp*^*2*^ = 0.63), but not for type (*F*_1,240_ = 1.87, *P* = 0.17, *ηp*^*2*^ = 0.007). On the other hand, a significant group-by-type interaction effect was found. Compared to HCs, participants with bvFTD performed significantly worse in both social and non-social stimulus types. The same pattern was observed in participants with AD. No significant differences were found in PD’s performance relative to HCs in either stimulus type (Table 2, Fig. 1C). The group-by-load interaction effect was also significant. Participants with bvFTD performed worse than HCs in all load levels (triplets, quartets, quintets). Similar results were observed in AD. PD participants presented significantly lower performance than HCs only in triplets (Table 2, Fig. S2). Finally, no significant load-by-type (*F*_2,480_ = 0.18, *P* = 0.83, *ηp*^*2*^ = 0.0007), neither group-by-type-by-load interaction effects were observed (*F*_6,480_ = 0.81, *P* = 0.56, *ηp*^*2*^ = 0.01).

### 3.2. Theta oscillatory correlates of social and non-social WM

**Fig. 2.**
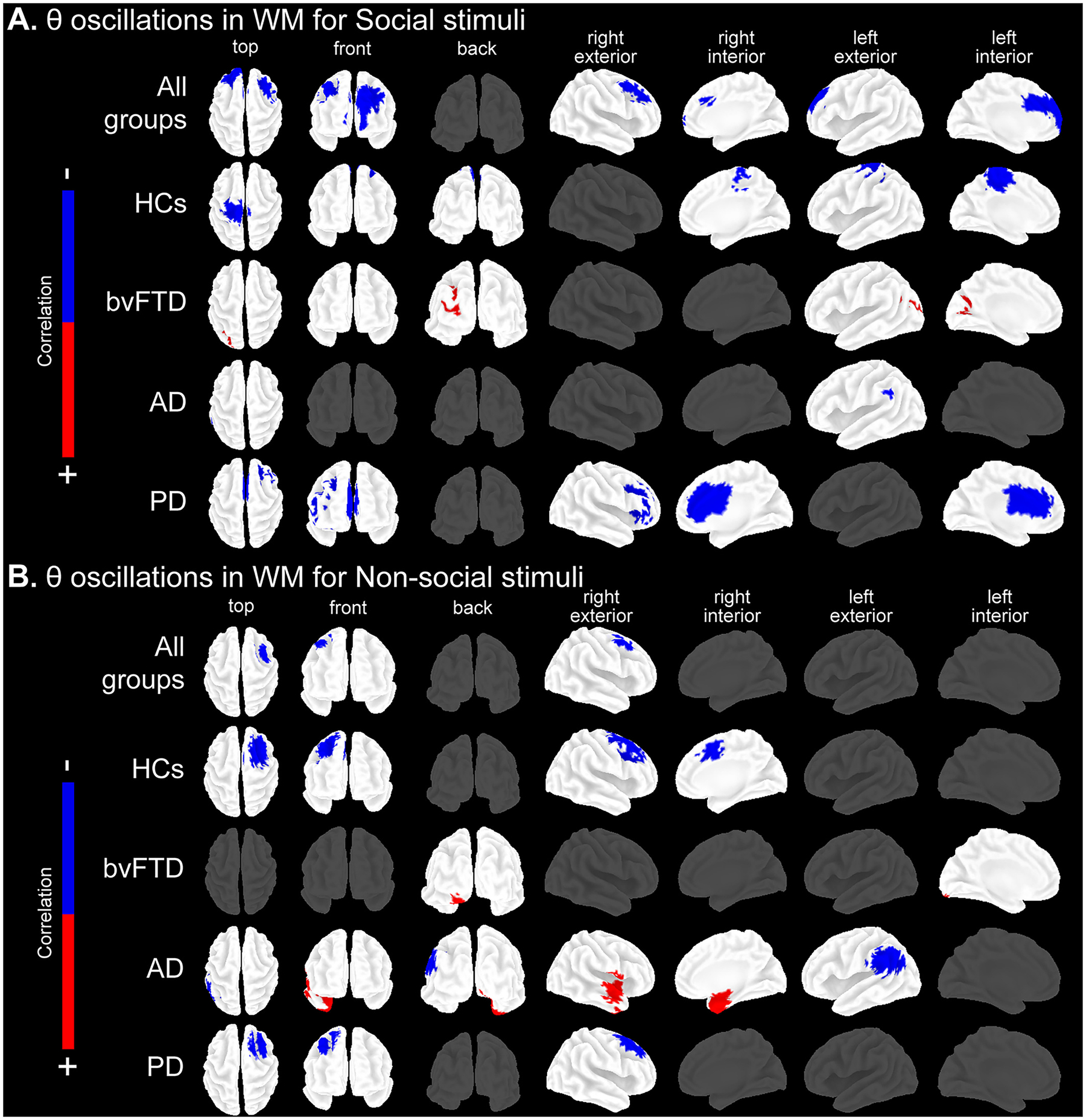
Associations between EEG θ oscillations and WM stimulus types. Pearson’s correlations between frequency-wise normalized current densities maps computed in the EEG θ frequency band, and WM performance (normalized IES) for social and non-social stimulus types (*P*≤ 0.05 FDR-corrected). Analyses were run in all groups together and individually per group (HCs, bvFTD, AD and PD). For further details see Table 3 and Table 4. Results are plotted in top, front, back, right external, right internal, left external, and left internal views of the brain. For results in high (quintets) and low (triplets) load levels see Fig. S3. Results were obtained with a demographically matched sample (Table S3.1). Associations between source space θ oscillations and WM for (A) social stimuli and (B) non-social stimuli. AD: Alzheimer’s disease, bvFTD: behavioral-variant frontotemporal dementia, HCs: healthy controls, IES: inverse efficiency score, PD: Parkinson’s disease.

In all groups together, better WM for social stimuli correlated to increased θ oscillations in extended bilateral frontocingulate areas (Table 3, Fig. 2). Better WM for non-social stimuli was associated with increased θ oscillations in a specific right frontal cluster (Table 4, Fig. 2). Also, better high load (quintets) WM was associated with increased bilateral frontocingulate and left parietal θ oscillations. Meanwhile, better low load (triplets) WM predominantly correlated with increased bilateral frontocingulate θ oscillations (Table S6.1, Fig. S3).

In HCs, better WM for social stimuli was associated with increased θ oscillations in bilateral frontocingulate and left parietal regions (Table 3, Fig. 2). In contrast, better WM for non-social stimuli was associated with increased θ oscillations in specific right frontocingulate areas (Table 4, Fig. 2). Moreover, better high load (quintets) WM was associated with increased θ oscillations in a specific right frontal cluster, while better low load (triplets) WM was associated with bilateral frontocingulate θ increment (Table S6.1, Fig. S3).

In bvFTD, WM deficits for social stimuli were associated with increased θ oscillations in left posterior regions (Table 3, Fig. 2), and WM deficits for non-social stimuli with a left posterior cluster (Table 4, Fig. 2). The same pattern was found among load levels, with significant associations between both high load (quintets) and low load (triplets) WM deficits, and increased θ oscillations in left posterior areas (Table S6.1, Fig. S3).

In AD, WM deficits for social stimuli were associated with decreased θ oscillations in the temporoparietal junction (Table 3, Fig. 2). In contrast, WM deficits for non-social stimuli were linked to decreased θ oscillations in extended temporoparietal areas, and to increased θ oscillations in right temporolimbic regions (Table 4, Fig. 2). High load (quintets) WM alterations were associated with decreased θ oscillations in left posterior regions, and low load (triplets) WM deficits correlated with a left parietal θ decrement (Table S6.1, Fig. S3).

In PD, we observed significant associations between better WM for social stimuli and increased θ oscillations in frontocingulate regions, while better WM for non-social stimuli was significantly associated with increased θ oscillations in a specific right frontal cluster (Table 3, Table 4, Fig. 2). Better high load (quintets) WM was associated with increased bilateral frontocingulate θ oscillations, while better low load (triplets) WM correlated with increased θ oscillations in a less extended bilateral frontocingulate cluster (Table S6.1, Fig. S3).

### 3.3. Brain network correlates of social and non-social WM

**Fig. 3.**
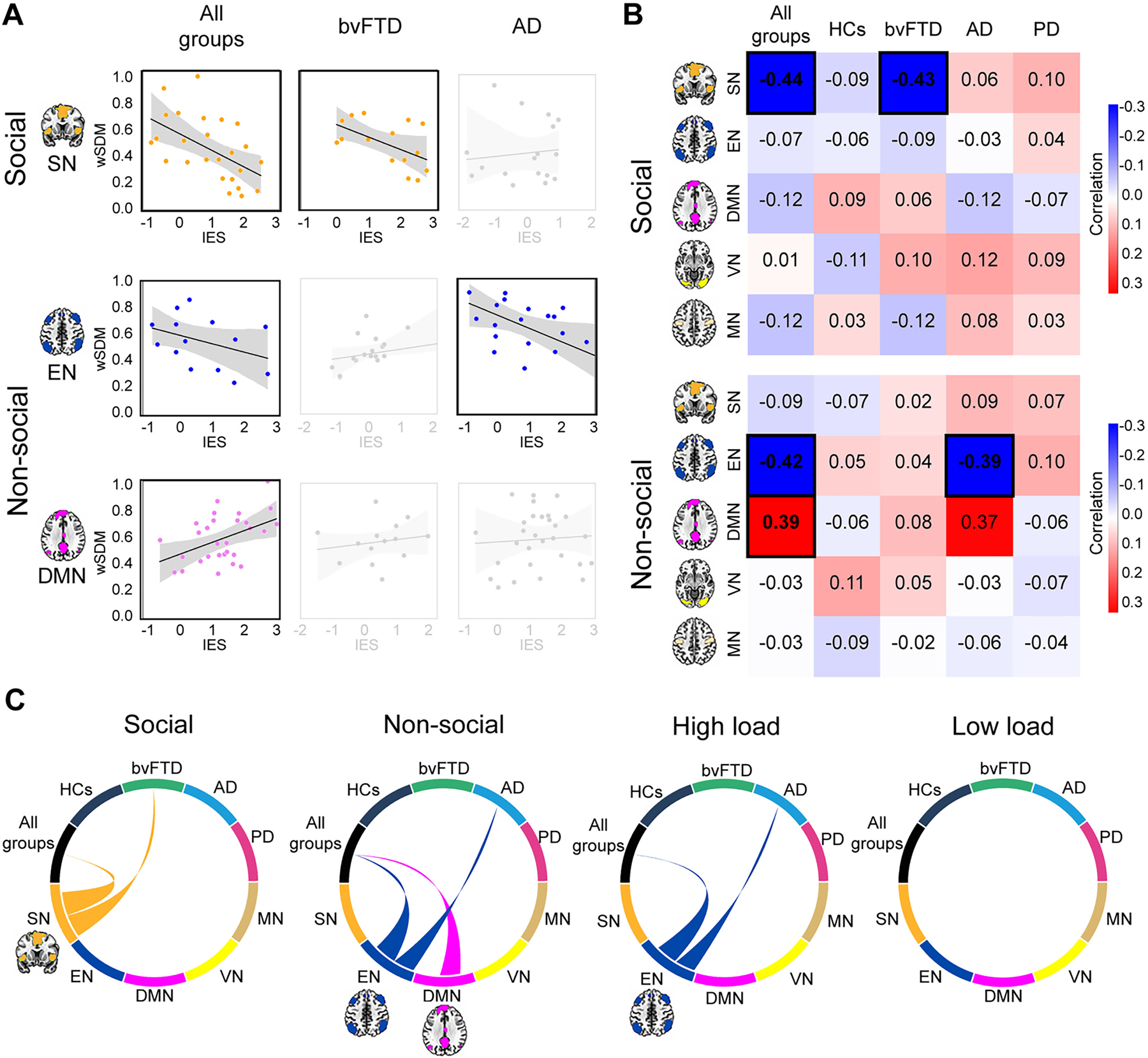
Associations between networks and WM. Seed analyses over five networks (SN, EN, DMN, VN, MN, *P*≤0.05 FDR-corrected) were performed to test the association between each network’ functional connectivity and WM performance (normalized IES) for social and non-social stimuli, and for high (quintets) and low (triplets) load levels. Analyses were run in all groups together and individually for HCs, bvFTD, AD and PD (Table 5). (A) Target network associations for stimulus type. Associations are plotted between target networks (SN, EN and DMN) and WM for social and non-social stimuli. WM for social stimuli was significantly linked to the SN in all groups together and in bvFTD. WM for non-social stimuli was significantly linked to the EN in all groups together and in AD, and to the DMN in all groups together. Black-border squares indicate significant associations. For HC and PD non-significant associations see Fig. S4. (B) Correlation matrix for each stimulus type. Correlation matrix for social and non-social stimulus type across all groups together and individual groups. Black-border squares indicate significant associations. (C) Functional connectivity associations for each stimulus type and load level. Circular plots represent all functional connectivity correlations between groups (upper semi-circle) and brain networks (lower semi-circles). Links represent significant group-network correlation. They are color-coded according to the network. High load (quintets) WM significantly correlated to the EN in all groups together and in AD. Non-significant results were found across associations with low load (for further details see Fig. S4). Results were obtained with a demographically matched sample (Table S3.2) and across scanners (Table S3.3). Standard masks (Shirer et al., 2012) were used to isolate the voxels involved in each network in MNI anatomical space. None of the participants showed head movements greater than 3 mm and/or rotations higher than 3° (Table S3.4). AD: Alzheimer’s disease, bvFTD: behavioral-variant frontotemporal dementia, DMN: default mode network, EN: executive network, HCs: healthy controls, IES: inverse efficiency score, MN: motor network, PD: Parkinson’s disease, SN: salience network, VN: visual network, wSDM: weighted Symbolic Dependence Metric.

For all groups together (Fig. 3), seed analyses revealed that the better the WM for social stimuli, the higher the SN connectivity (*r* = −0.443, *P-FDR* = 0.03). In contrast, better WM for non-social stimuli was significantly associated with increased EN (*r* = −0.424, *P-FDR* = 0.03) and decreased DMN (*r* = 0.387, *P-FDR* = 0.04) connectivity. Also, better high load (quintets) WM was associated with increased EN connectivity (*r* = −0.401, *P-FDR* = 0.05). No significant associations were found for control networks (VN and MN) neither with low load (triplets) (see Table 5, Fig. S4). HCs presented non-significant associations.

In bvFTD, WM deficits for social stimuli were associated to reduced SN connectivity (*r* = −0.431, *P-FDR* = 0.03). In AD, significant associations were found between WM deficits for non-social stimuli (*r* = −0.395, *P-FDR* = 0.04) and for high load (quintets) level (*r* = −0.372, *P-FDR* = 0.04) and reduced EN connectivity. PD presented non-significant associations. Finally, non-significant results were found in any individual group for neither low load (triplets) associations (Table 5, Fig. S4).

## 4. Discussion

We examined the WM domain-specificity for social vs non-social stimuli in healthy participants and neurodegenerative conditions at behavioral, oscillatory, and functional connectivity levels. In HCs, a WM load effect and decreased WM for social stimuli confirmed our hypotheses. Considering all groups together, WM for social stimuli was associated with higher frontocingulate θ oscillations and SN connectivity. Conversely, WM for non-social stimuli was linked to canonical right frontal θ oscillations, higher EN and lower DMN connectivity. Relative to HCs, bvFTD presented generalized WM deficits associated with increased θ oscillations in posterior regions (being abolished in canonical frontal hubs), and WM for social stimuli specifically linked to lower SN connectivity. In AD, generalized WM deficits were related to temporoparietal θ oscillations, with WM for non-social stimuli particularly linked to lower EN connectivity. PD showed preserved WM, with social and non-social stimuli related to frontocingulate and frontal θ oscillations, respectively. Together, these multimodal findings reveal specific social and non-social stimuli mechanisms in WM across different pathophysiological models sensitive to WM and social processing impairments (bvFTD), generalized cognitive deficits (AD), and unspecific alterations (PD).

### 4.1. Domain-specific WM for social stimuli in controls

In HCs, behavioral and brain correlates confirmed the task’s robustness and our predictions. WM performance was reduced by high load and social stimuli (social impairment effect) (Cowan, 2017; Garrison and Schmeichel, 2019; Plancher et al., 2019). WM social modulation increased θ oscillations in extended regions beyond canonical hubs (Dai et al., 2017; Pavlov and Kotchoubey, 2020), including: frontal [involved in action-execution (Gu et al., 2019), WM (Marvel et al., 2019), and social-saliency processing (Van der Molen et al., 2017)]; cingulate [related to error-prediction and ‘other-oriented’ cues (Apps et al., 2016; Billeke et al., 2013)]; and left parietal [engaged in social processing (Billeke et al., 2014) and verbal WM (Eriksson et al., 2015; Li et al., 2017)] hubs. This broader network suggests θ oscillations are involved in the integration of bottom-up (stimulus-driven) and top-down (cognitive) processes across distributed areas (Brenner et al., 2014). The need for more efficient propagation of information in order to cope with socially-demanding WM processes might recruit this extended oscillatory network. Regarding rsfMRI, WM for social stimuli was related to SN connectivity. Insular-cingulate hubs (Seeley, 2019; Seeley et al., 2007) are critical for executive and social processing (Uddin, 2015) by mediating the interaction between bottom-up and top-down WM processes for social stimuli (Luo et al., 2014). Then, the SN seems to be critical to successful WM for externally-perceived social stimuli. This network would dynamically reallocate resources between the DMN (related to social cognition) and the EN (linked to WM) to optimize responses to salient (social) stimuli (Maehara, 2017; Uddin, 2015). In contrast, WM for non-social stimuli involved the predicted EN-DMN anticorrelation (Chai et al., 2018; Liang et al., 2016). Moreover, the lack of associations with control networks (VN and MN) confirmed the differential impact of social and non-social stimuli over WM. All in all, results in HCs and in all groups together support a relative domain-specific WM for social stimuli with increased behavioral demands indexed by frontocinguloparietal θ oscillations and the SN connectivity, compared to frontal θ oscillations and EN-DMN anticorrelation for non-social stimuli.

### 4.2. Distinct WM mechanisms across neurodegenerative models

In bvFTD, contrarily to the social stimuli selectivity hypothesis, WM deficits were generalized. Although to some extent unexpected, this is consistent with the syndrome’s well-known, overall, and *sui generis* dysexecutive profile (Beeldman et al., 2018; Gonzalez-Gomez et al., 2021; Kamath et al., 2019). Similarly, overall absent frontal θ correlates suggest a generalized WM alteration linked to impaired oscillatory mechanisms. Indeed, WM decay was linked to higher posterior visual-encoding θ oscillations (Takase et al., 2022), interpreted as a paradoxical response to frontal θ abolition (Caso et al., 2012; Metin et al., 2018) necessary for WM compensation (Dai et al., 2017). Beyond bvFTD lack of domain-specificity across behavioral and oscillatory correlates, impaired WM for social stimuli was specifically linked to lower SN connectivity. This network is distinctly disrupted in bvFTD relative to HCs and AD (Brown et al., 2020; Moguilner et al., 2021; Pini et al., 2022; Salamone et al., 2021; Seeley, 2019; Zhou et al., 2010) and has been related to their inability to hold and manipulate social cues in complex social situations (Baez et al., 2014; Zhou and Seeley, 2014). Then, SN abnormalities may disrupt the ‘switch’ between the EN and DMN during executive processes involving social stimuli (Maehara, 2017; Uddin, 2015). Altogether, a primary (domain-neutral), *sui generis* WM impairment in bvFTD is accompanied by a distinctive social domain-specific WM pathophysiological mechanism related to the SN.

In AD, results supported the hypothesis of unspecific WM decline across behavioral and brain correlates, possibly explained by overall cognitive deficits (Ibáñez et al., 2021b; Ramanan et al., 2017b). Resulting socially-related temporoparietal θ decay confirmed the DMN-related attentional and social processes engagement (Igelström and Graziano, 2017) already impaired in AD (Lattanzio et al., 2021). Moreover, non-social stimuli deficits were linked to broader temporoparietal θ decrease (Goodman et al., 2019; Kobylecki et al., 2018) and to unexpected temporolimbic θ increase. Recent works in AD have reported both positive and negative θ dynamics, in terms of hypo- and hyperconnectivity (Herzog et al., 2022; Prado et al., 2023). The observed mixed correlations may be explained by temporo-posterior atrophy and decreased dynamic range of cortical activity (Prado et al., 2023). Positive θ associations could be triggered by functional reorganization (Parra et al., 2017) including temporolimbic θ hyperconnectivity (Prado et al., 2023), and overall cognitive deficits (Musaeus et al., 2018). Finally, canonical frontal θ correlates were absent during both stimulus types, previously related to AD cognitive withdrawal (Hata et al., 2016). Regarding rsfMRI, the observed link between non-social WM deficits and lower EN connectivity adds evidence of a disrupted frontoparietal mechanism (Zhao et al., 2019) underlying this condition’ WM decay (Wang et al., 2015). In contrast to bvFTD, AD social deficits appear to be related to general-cognitive alterations. Thus, results support the overall WM alteration hypothesis in AD, primarily indexed by temporoparietal θ and EN dysfunctions that jointly aligns with a domain-general WM pathophysiological mechanism.

In PD, the predicted spared WM suggests a cognitive compensation possibly related to dopaminergic medication (Moustafa et al., 2013; Salmi et al., 2020). Social modulation over WM was comparable to HCs, indicating that WM for social stimuli is not primarily altered in this disease. In fact, social processing deficits in PD might be mediated by general executive functions (Kosutzka et al., 2019; Maggi et al., 2022; Romosan et al., 2019). Similarly, preserved frontocingulate and frontal θ correlates in both stimulus type can also be explained by the overall-brain θ regularization triggered by dopaminergic medication (Moustafa et al., 2013; Orcioli-Silva et al., 2020; Ramos and Machado, 2021; Simioni et al., 2017). Lack of neurofunctional correlates (rsfMRI) is not surprising considering the more lenient impact of atrophy, the relative cognitive preservation, and the ‘ON’ dopaminergic-medication state of our sample (Cole et al., 2013; Dang et al., 2012; Kalpouzos et al., 2012; Wolters et al., 2019). In fact, absent connectivity correlates of executive performance in PD is not novel (Engels et al., 2018). Multimodal results converge in a spared dopaminergic-medicated PD model with predominant frontal θ correlates supporting WM and social processing preservation.

### 4.3. Theoretical and clinical implications

Our findings support theoretical accounts of a relative domain-specific WM for social stimuli, with higher behavioral demands and extended social brain regions beyond canonical WM hubs. The cingulate cortex was engaged in all domain-specific neurofunctional mechanisms: the distributed θ oscillations and the SN. This hub may play a pivotal role during WM for social stimuli. Specifically, it may index error-prediction WM mechanisms that are particularly modulated by the integration of socio-contextual information (Lavin et al., 2013). Then, when social and cognitive processes operate simultaneously and compete with each other for the limited neural resources (Hur et al., 2017), the cingulate may coordinate their interaction allowing the successful maintenance and manipulation of social cues. This interpretation fits well with externally-perceived social stimuli, contrasting other WM reports based on internally-generated social cues (Meyer and Lieberman, 2012; Meyer et al., 2012; Meyer et al., 2015). Our findings support the idea that previous controversies regarding WM domain-specificity (Meyer and Collier, 2020; Thornton and Conway, 2013; Xin and Lei, 2015) are primarily explained by differences in the internal vs external stimulus sources (Smith et al., 2017).

The detection of specific WM mechanisms for social stimuli carries clinical implications providing better characterization of neurodegenerative conditions. Comparable bvFTD and AD behavioral deficits were indexed by different domain-specific and -general pathophysiological mechanisms, respectively. Then, findings suggest differential therapeutic strategies, including: (a) general-WM training as a potential tool to induce EN enhancement (Constantinidis and Klingberg, 2016) and to improve daily-functioning in AD (Hernes et al., 2021); and (b) both general- and social specific-WM training to boost everyday social competence in bvFTD (Maehara, 2017; Meyer and Lieberman, 2012). Moreover, spared neurocognitive mechanisms in PD highlight the essential role of dopaminergic medication in WM preservation beyond striatal alterations. In sum, we offer relevant evidence of distinct neural correlates underlying social modulation over WM across neurodegenerative models, allowing differential diagnosis and treatment.

### 4.4. Limitations and further research

We acknowledge certain limitations to our study and outline new avenues for further research. First, although our work is based on a modest sample size, it is larger than those of other multimodal neurodegenerative reports (Birba et al., 2021; Garcia-Cordero et al., 2016; Hughes et al., 2011; Legaz et al., 2022; Melloni et al., 2016; Moretti et al., 2009; Salamone et al., 2021). Moreover, strict control of demographic and clinical variables, as well as systematic diagnostic procedures, counteract this limitation. Also, our sample size power analysis, the results consistency across dimensions together with moderate-to-large effect sizes further attests to their robustness. In any case, future studies should replicate and extend these results with larger samples. Second, we focused on θ oscillations since research strongly supports its involvement in WM (Pavlov and Kotchoubey, 2020) across neurodegeneration (Goodman et al., 2019), and social processing (Gregory et al., 2021; van der Velde et al., 2021). However, future works should also target alpha/gamma oscillations and cross-frequency coupling also related to WM (Dai et al., 2017; Roux and Uhlhaas, 2014) and reported impaired in neurodegenerative conditions (Güntekin et al., 2022; Ishii et al., 2017; Kitchigina, 2018). Third, beyond the novel contributions of convergent rsEEG and rsfMRI methodologies across multiple neurodegenerative models, future works should also include active paradigms to better elucidate brain networks directly engaged in social WM modulation. Finally, our work rises new evidence regarding the domain-specificity of a critical daily-life process (Maresca et al., 2020; Porcelli et al., 2019). Our findings call for a more synergic understanding of social cognition and WM blending. These processes are not isolated, but integrated across different dimensions. New studies should examine how WM for social stimuli differentially impacts everyday functioning across neurodegenerative profiles with an ecological approach. This would better capture implicit socio-contextual modulations over WM and social cognition dynamics in real-life settings (Ibáñez, 2022).

## 5. Conclusions

Our multimodal neurodegenerative lesion model approach reveals convergent evidence of social and non-social effects over WM across healthy controls and neurodegenerative conditions. Findings support a relative domain-specific WM for social stimuli indexed by frontocinguloparietal θ oscillations and the SN that contrast with canonical frontal θ correlates and EN-DMN anticorrelation for non-social stimuli. Also, results provide different pathophysiological mechanisms, including a bvFTD primary WM alteration but specific network mechanisms linked to WM for social stimuli, domain-general WM deficits linked to cognitive deficits and related pathological brain correlates in AD, and behavioral and neurofunctional preservation in dopaminergic-medicated PD. Further research may bring a new clinical agenda favoring differential diagnosis and treatment among neurodegenerative conditions with common WM and social processing alterations.

## Supplementary Material

1

## Figures and Tables

**Table 1:** Samples’ demographic and neurocognitive data.

	HCs (*n* = 90)	bvFTD (*n* = 42)	AD (*n* = 54)	PD (*n* = 59)	Stats	Post-hoc comparisons

Demographics						
Sex (M:F)	39:51	26:16	22:32	35:24	χ^2^ = 7.87, *P* = 0.048*	HCs-bvFTD: *P* = 0.071; HCs-AD: *P* = 0.896; HCs-PD: *P* = 0.081
Age ^a^	71.21 (6.82)	69.45 (9.67)	74.65 (5.82)	67.9 (9.01)	*F* = 7.69, *P* < 0.001*, *ηp*^2^ = 0.08	HCs-bvFTD: *P* = 0.618; HCs-AD: *P* = 0.051; HCs-PD: *P* = 0.054
Education	13.4 (3.92)	13.98 (4.61)	11.7 (4.82)	11.8 (4.7)	*F* = 3.61, *P* = 0.013*, *ηp*^2^ = 0.04	HCs-bvFTD: *P* = 0.899; HCs-AD: *P* = 0.120; HCs-PD: *P* = 0.138
Handedness (R:L)	85:1	35:1	50:1	56:2	–	–
Cognitive assessment						
MoCA ^a^	25.72 (3.15)	20.69 (4.94)	17.27 (4.38)	23.33 (4.44)	*F* = 50.22, *P* < 0.001*, *ηp*^2^ = 0.39	HCs-bvFTD: *P* < 0.001*; HCs-AD: *P* < 0.001*; HCs-PD: *P* = 0.003*
IFS ^a^	22.08 (3.69)	18.81 (5.26)	14.95 (4.98)	19.21 (4.85)	*F* = 27.39, *P* < 0.001*, *ηp*^2^ = 0.26	HCs-bvFTD: *P* = 0.001*; HCs-AD: *P <* 0.001*; HCs-PD: *P* = 0.001*

Results are presented as mean (*SD*). The asterisk (*) indicates significant differences with an alpha level of *P* < 0.05.

a indicates variables with significant differences (*P* < 0.05) between neurodegenerative groups, precluding comparisons between them in our target measures. Demographic and cognitive data were assessed through ANOVAs and Tukey post-hoc pairwise comparisons –except for sex, which was analyzed via Pearson’s chi-squared (χ2) test. Effects sizes were calculated through partial eta (*ηp*^2^). AD: Alzheimer’s disease, bvFTD: behavioral-variant frontotemporal dementia, HCs: healthy controls, IFS: INECO Frontal Screening, MoCA: Montreal Cognitive Assessment, PD: Parkinson’s disease.

**Table 2:** Statistical comparison between group * load * stimulus type.

Group-by-type						

Stimulus type	HCs	bvFTD	AD	PD	Stats	Post-hoc comparisons
Social	− 0.31 (0.84)	0.23 (0.99)	0.52 (0.97)	− 0.05 (0.93)	*F_3_*_,240_ = 4.99, *P* = 0.002*, *ηp^2^* = 0.06	HCs-bvFTD: *P* = 0.001* HCs-AD: *P <* 0.001* HCs-PD: *P* = 0.10
Nonsocial	− 0.44 (0.92)	0.35 (0.98)	0.53 (0.96)	− 0.19 (0.95)		HCs-bvFTD: *P <* 0.001* HCs-AD: *P* < 0.001* HCs-PD: *P* = 0.23

Group-by-load						

Load level	HCs	bvFTD	AD	PD	Stats	Post-hoc comparisons

Triplets	− 1.04 (0.76)	− 0.19 (1.04)	0.1 (1.07)	− 0.59 (0.92)	*F*_6,480_ = 7.28, *P* < 0.001*, *ηp^2^* = 0.08	HCs-bvFTD: *P* < 0.001* HCs-AD: *P* < 0.001* HCs-PD: *P* = 0.01*
Quartets	− 0.42 (0.67)	0.21 (0.92)	0.56 (0.89)	− 0.26 (0.83)		HCs-bvFTD: *P* < 0.001* HCs-AD: *P* < 0.001* HCs-PD: *P* = 0.69
Quintets	0.35 (0.58)	0.85 (0.68)	0.92 (0.72)	0.5 (0.71)		HCs-bvFTD: *P* < 0.001* HCs-AD: *P* < 0.001* HCs-PD: *P* = 0.41

Results are presented as mean (*SD*). The asterisk (*) indicates significant differences with an alpha level of *P* < 0.05. Between-group comparison on WM performance (normalized inverse efficiency score [IES]) for stimulus type (social, non-social) and load level (triplets, quartets, quintets) was assessed through a mixed model ANOVA (type III) and Tukey post-hoc comparisons. Effects sizes were calculated through partial eta (*ηp^2^*). Results are plotted in Fig. 1C. AD: Alzheimer’s disease, bvFTD: behavioral-variant frontotemporal dementia, HCs: healthy controls, PD: Parkinson’s disease.

**Table 3:** Associations between EEG θ oscillations and WM for social stimuli.

Regions	*r*	*P-FDR*	MNI coordinates			BA
			x	y	z	

Social						
All groups together						
Superior frontal gyrus R	− 0.528	0.041	25	35	45	8
Superior frontal gyrus L	− 0.525	0.044	− 10	− 55	40	9
Middle frontal gyrus R	− 0.529	0.041	45	20	50	8
Middle frontal gyrus L	− 0.527	0.042	− 25	45	35	9
Medial orbital frontal L	− 0.527	0.042	− 5	− 65	0	10
Medial superior frontal gyrus L	− 0.527	0.042	− 10	− 60	30	10
Inferior frontal (pars opercularis) R	− 0.527	0.042	50	20	40	8
Anterior cingulum L	− 0.527	0.042	− 10	35	30	8
Middle cingulum L	− 0.527	0.042	− 5	− 20	35	8
HCs						
Superior frontal gyrus R	− 0.556	0.044	20	− 10	− 60	6
Superior frontal gyrus L	− 0.551	0.046	− 25	10	60	6
Middle frontal gyrus L	− 0.551	0.046	− 25	− 15	− 50	6
Supplementary motor area R	− 0.558	0.041	5	− 25	60	6
Supplementary motor area L	− 0.583	0.045	− 15	− 15	60	6
Precentral gyrus L	− 0.589	0.041	− 25	− 20	60	6
Parahippocampal gyrus L	− 0.601	0.039	− 20	− 25	− 20	36
Middle cingulum L	− 0.589	0.041	− 10	− 25	50	6
Paracentral lobe R	− 0.556	0.044	5	− 25	65	4
Paracentral lobe L	− 0.600	0.039	− 15	− 30	60	4
Postcentral gyrus L	− 0.597	0.040	− 20	− 30	55	1
Precuneus L	− 0.641	0.035	− 15	− 35	60	7
Superior parietal lobe L	− 0.553	0.045	− 20	− 40	65	1
BvFTD						
Cuneus L	0.707	0.029	− 15	− 75	20	18
Calcarine fissure L	0.722	0.027	− 20	− 70	20	19
Fusiform gyrus L	0.729	0.027	− 25	− 75	20	19
Superior occipital gyrus L	0.728	0.027	− 20	− 75	20	19
Middle occipital gyrus L	0.709	0.029	− 30	− 80	20	19
Fusiform gyrus L	0.727	0.027	− 20	− 80	20	18
Lingual gyrus L	0.699	0.030	− 15	− 85	15	18
AD						
Supramarginal gyrus L	− 0.550	0.044	− 65	− 45	30	39
Inferior parietal lobe L	− 0.541	0.044	− 60	− 50	40	40
Angular gyrus L	− 0.532	0.045	− 60	− 55	35	40
PD						
Inferior frontal (pars triangularis) R	− 0.792	0.021	40	35	15	46
Medial superior frontal gyrus R	− 0.778	0.022	5	30	40	6
Superior frontal gyrus R	− 0.815	0.019	20	30	30	9
Middle frontal gyrus R	− 0.805	0.019	35	25	35	9
Inferior frontal (pars opercularis) R	− 0.810	0.019	35	20	35	9
Insula R	− 0.799	0.021	30	20	15	45
Anterior cingulum R	− 0.840	0.016	5	10	30	24
Anterior cingulum L	− 0.837	0.017	− 5	10	25	24
Middle cingulum R	− 0.832	0.017	5	10	25	32
Middle cingulum L	− 0.796	0.020	− 5	10	35	32
Supplementary motor area R	0.789	0.021	15	5	45	6

Pearson correlations (*P* ≤ 0.05 FDR-corrected) were performed to test the association between normalized current density maps in the EEG θ frequency band and WM performance (normalized inverse efficiency score [IES]), for social stimulus type. Analyses were run in all groups together and individually per group (HCs, bvFTD, AD and PD). Results are plotted in Fig. 2.A. These results were obtained with a demographically matched sample (see Table S3.1). AD: Alzheimer’s disease, BA: Brodmann area, bvFTD: behavioral-variant frontotemporal dementia, HCs: healthy controls, PD: Parkinson’s disease.

**Table 4:** Associations between EEG θ oscillations and WM for non-social stimuli.

Regions	*r*	*P-FDR*	MNI coordinates			BA
			x	y	z	

Non-social						
All groups together						
Superior frontal gyrus R	− 0.525	0.044	30	5	65	6
Middle frontal gyrus R	− 0.527	0.042	45	10	55	8
Precentral gyrus R	− 0.525	0.044	45	− 5	60	6
HCs						
Superior frontal gyrus R	− 0.672	0.032	3	51	8	6
Frontal inferior operc. R	− 0.626	0.037	35	15	35	8
Middle frontal gyrus R	− 0.685	0.031	25	15	50	8
Middle cingulum R	− 0.641	0.035	15	10	40	8
Supplementary motor area R	− 0.656	0.034	15	5	45	6
Precentral gyrus R	− 0.660	0.034	30	− 5	50	6
BvFTD						
Calcarine fissure L	0.623	0.037	− 10	− 100	− 10	18
Lingual gyrus L	0.671	0.032	− 10	− 100	− 15	18
AD						
Rolandic operculum R	0.552	0.045	50	5	0	44
Insula R	0.558	0.044	40	5	− 10	13
Inferior temporal gyrus R	0.567	0.043	35	5	− 45	38
Caudate R	0.565	0.043	35	5	− 15	38
Fusiform gyrus R	0.575	0.042	25	5	− 45	38
Middle temporal pole R	0.567	0.042	25	5	− 35	36
Superior temporal pole R	0.572	0.041	25	5	− 20	34
Amygdala R	0.577	0.041	20	0	− 15	38
Parahippocampal gyrus R	0.572	0.041	15	0	− 15	36
Postcentral gyrus R	− 0.553	0.045	65	− 5	15	4
Superior temporal gyrus R	− 0.563	0.043	65	− 5	0	21
Superior temporal gyrus L	− 0.579	0.041	− 55	− 45	20	22
Hippocampus R	0.570	0.042	15	− 5	− 15	28
Middle frontal gyrus L	− 0.551	0.045	− 25	− 15	− 50	6
Inferior parietal lobe L	− 0.616	0.038	− 60	− 50	40	39
Supramarginal gyrus L	− 0.627	0.037	− 60	− 50	− 35	39
Middle temporal gyrus L	− 0.579	0.041	− 50	− 50	20	39
Angular gyrus L	− 0.616	0.038	− 60	− 55	35	39
PD						
Medial superior frontal gyrus R	− 0.621	0.038	5	40	55	8
Superior frontal gyrus R	− 0.606	0.039	15	30	60	6
Supplementary motor area R	− 0.600	0.039	10	25	60	6

Pearson correlations (*P* ≤ 0.05 FDR-corrected) were performed to test the association between normalized current density maps in the EEG θ frequency band and WM performance (normalized inverse efficiency score [IES]), for non-social stimulus type. Analyses were run in all groups together and individually per group (HCs, bvFTD, AD and PD). Results are plotted in Fig. 2.B. These results were obtained with a demographically matched sample (see Table S3.1). AD: Alzheimer’s disease, BA: Brodmann area, bvFTD: behavioral-variant frontotemporal dementia, HCs: healthy controls, PD: Parkinson’s disease.

**Table 5:** Associations between functional connectivity networks and WM.

Network	Social		Non-social		High load (quintets)		Low load (triplets)	
	*r*	*P-FDR*	*r*	*P-FDR*	*r*	*P-FDR*	*r*	*P-FDR*

All groups together								
SN	− 0,443	0,036*	− 0,090	0,216	0,037	0,554	− 0,025	0,848
EN	− 0,068	0,297	− 0,424	0,037*	− 0,401	0,050*	− 0,068	0,286
DMN	− 0,121	0,160	0,387	0,041*	0,035	0,571	0,048	0,416
VN	0,013	0,536	− 0,035	0,558	− 0,122	0,164	− 0,062	0,320
MN	− 0,123	0,165	− 0,033	0,589	− 0,088	0,222	0,116	0,174
HCs								
SN	− 0,094	0,219	− 0,069	0,282	− 0,120	0,160	− 0,049	0,434
EN	− 0,060	0,323	0,049	0,418	− 0,109	0,188	− 0,033	0,580
DMN	0,092	0,216	− 0,061	0,322	0,031	0,648	− 0,063	0,311
VN	− 0,113	0,175	0,116	0,177	0,094	0,230	− 0,043	0,459
MN	0,033	0,609	− 0,090	0,227	0,042	0,399	− 0,040	0,485
BvFTD								
SN	− 0,431	0,038*	0,021	0,951	− 0,123	0,161	0,092	0,215
EN	− 0,092	0,217	0,044	0,459	− 0,019	0,448	− 0,113	0,179
DMN	0,057	0,351	0,077	0,266	− 0,071	0,277	0,033	0,603
VN	0,102	0,196	0,047	0,425	0,056	0,358	0,107	0,183
MN	− 0,121	0,165	− 0,025	0,764	0,027	0,742	0,104	0,199
AD								
SN	0,057	0,357	0,095	0,214	0,022	0,904	− 0,116	0,176
EN	− 0,032	0,650	− 0,395	0,041*	− 0,372	0,046*	0,119	0,169
DMN	− 0,117	0,178	0,375	0,064	− 0,089	0,223	0,080	0,254
VN	0,120	0,168	− 0,034	0,586	0,094	0,213	− 0,063	0,310
MN	0,080	0,258	− 0,064	0,316	0,017	0,974	− 0,055	0,356
PD								
SN	0,098	0,209	0,074	0,276	− 0,033	0,585	− 0,073	0,272
EN	0,039	0,524	0,103	0,193	0,102	0,196	0,089	0,222
DMN	− 0,073	0,271	− 0,065	0,307	0,122	0,169	0,035	0,579
VN	0,088	0,227	− 0,068	0,285	0,093	0,216	0,105	0,195
MN	0,035	0,574	− 0,045	0,432	0,032	0,649	0,119	0,168

Seed analyses over five networks (SN, EN, DMN, MN, VN, *P* ≤ 0.05 FDR-corrected) were performed to test the association between the functional connectivity of each network and WM performance (normalized inverse efficiency score [IES]), for social and non-social stimulus types, and the most extreme load level (quintets [high load] and triplets [low load]) conditions. Analyses were run in all groups together and individually per group (HCs, bvFTD, AD and PD). The asterisk (*) indicates significant association with an alpha level of *P-FDR* ≤ 0.05. Results are plotted in Fig. 3. and in Fig. S4. These results were obtained with a demographically matched sample (see Table S3.2). Standard masks were used to isolate the voxels that are typically involved in each resting-state network, based on the MNI anatomical space. None of the participants showed head movements greater than 3 mm and/or rotations higher than 3^◦^ (see Table S3.4). AD: Alzheimer’s disease, bvFTD: behavioral-variant frontotemporal dementia, DMN: default mode network, EN: executive network, HCs: healthy controls, MN: motor network, PD: Parkinson’s disease, SN: salience network, VN: visual network.

## Data Availability

The datasets generated during and/or analysed during the current study are available in the OSF repository, http://osf.io/bx27h (Legaz, 2022).

## Abbreviations:

AD: Alzheimer’s disease
BvFTD: behavioral variant frontotemporal dementia
HCs: healthy controls
IES: inverse efficiency score
DMN: default mode network
EN: executive network
MN: motor network
NINCDS-ADRDA: National Institute of Neurological and Communicative Disorders and Stroke - Alzheimer’s Disease and Related Disorders Association
PD: Parkinson’s disease
rsEEG: resting-state EEG
rsfMRI: resting-state functional magnetic resonance imaging
sLORETA: standardized Low-Resolution Electromagnetic Tomography method
SN: salience network
SPM12: Statistical Parametric Mapping software v.12
θ: theta
VN: visual network
WM: working memory
