# Supplementary material for: Social and non-social working memory in neurodegeneration
Source: Neurobiol Dis. Author manuscript; Available in PMC 2024 Jun 14. (PMC11177282; doi:10.1016/j.nbd.2023.106171)
Supplement: 1 [file NIHMS1996710-supplement-1.docx]

Supplementary data

[1. Material S1. Sample characterization 2](#_Toc134437277)

[1.1. Material S1.1. Sample size and power estimation 2](#_Toc134437278)

[1.2. Table S1.2. Gray matter atrophy patterns in patients 2](#_Toc134437279)

[2. Material S2. Task design 3](#_Toc134437280)

[2.1. Material S2.1. Behavioral task validation 3](#_Toc134437281)

[2.1. Table S2.1.1. Stimuli lexical validation 4](#_Toc134437282)

[2.2. Table S2.1.2. Stimuli validation per type 5](#_Toc134437283)

[2.3. Table S2.1.3. Levenshtein distance between old/new words 5](#_Toc134437284)

[3. Material S3. EEG / fMRI acquisition and preprocessing 5](#_Toc134437285)

[3.1. Table S3.1. EEG subsamples’ demographic data 5](#_Toc134437286)

[3.2. Table S3.2. fMRI subsamples’ demographic data 6](#_Toc134437287)

[3.3. Table S3.3. Specific neuroimaging parameters per scanner 6](#_Toc134437288)

[3.4. Table S3.4. fMRI movement’ parameters 8](#_Toc134437289)

[4. Material S4. Behavioral data analysis 8](#_Toc134437290)

[4.1. Table S4.1. Final number of trials per group * type * load 8](#_Toc134437291)

[4.2. Table S4.2. Mean final number of trials among groups 9](#_Toc134437292)

[4.3. Table S4.3. Shapiro-Wilk test for normality 9](#_Toc134437293)

[4.4. Table S4.4. Levene test for homoscedasticity 9](#_Toc134437294)

[4.5. Table S4.5. Individual groups' IES comparison between type and load 9](#_Toc134437295)

[5. Material S5. EEG and neuroimaging statistical analysis 11](#_Toc134437296)

[5.1. Material S5.1. EEG: source localization analysis 11](#_Toc134437297)

[5.2. Material S5.2. Neuroimaging: functional connectivity analysis 11](#_Toc134437298)

[6. Material S6. EEG θ oscillations results 12](#_Toc134437299)

[6.1. Table S6.1. Associations between EEG θ oscillations and WM load level 12](#_Toc134437300)

[Supplementary Figures 14](#_Toc134437301)

[Figure S1. Trial’s minimum threshold of 250 ms 14](#_Toc134437302)

[Figure S2. Behavioral results: in-group comparisons among type and load 16](#_Toc134437303)

[Figure S3. Associations between EEG θ oscillations and WM load level 17](#_Toc134437304)

[Figure S4. Associations between functional connectivity networks and WM 18](#_Toc134437305)

# 1. Material S1. Sample characterization

## 1.1. Material S1.1. Sample size and power estimation

To determine the sample size required for our study, we ran a power estimation analysis on G*Power 3.1 (Faul et al., 2007). Given our statistical design (based on a 4*2*3 mixed effects ANOVA, within-between interaction), we considered the following parameters: an small-to-medium effect size of *f* = 0.15 (Cohen, 2013); an alpha level of *P =* 0.05; a power of 0.80 –higher than the median of powers analyzed in previous studies in the area for detecting large effect sizes (Szucs and Ioannidis, 2017); four manipulation groups (HCs, bvFTD, AD and PD); and six measurements (stimulus type [social, non-social] * load level [triplets, quartets, quintets]). This analysis showed that a total sample size of *n =* 76 is adequate to detect the estimated effects. A post-hoc power estimation confirmed that this assumption was met, since the final sample size of our study *n =* 245 reaches a power of 0.99. Power analyses confirmed the adequacy of our sample size.

## 1.2. Table S1.2. Gray matter atrophy patterns in patients

| **Regions** | **Cluster** | | **Peak** | **MNI coordinates** | | |
| --- | --- | --- | --- | --- | --- | --- |
|  | **N° voxels** | ***P-FWE*** | ***t*** | **x** | **y** | **z** |
| **bvFTD Atrophy** | | | | | | |
| Inferior Temporal Gyrus R | 18290 | 0,0012 | 5,82 | 48 | -18 | -20 |
| Middle Temporal Gyrus R |  |  | 5,45 | 44 | -2 | -29 |
| *Insula R* |  |  | 3,62 | 27 | 20 | -13 |
| *Inferior Orbitofrontal Gyrus R* |  |  | 4,6 | 26 | 34 | -11 |
| *Middle Orbitofrontal Gyrus R* |  |  | 4,39 | 25 | 39 | -11 |
| Hippocampus L | 7426 | 0,0038 | 5,2 | -32 | -8 | -20 |
| Inferior Temporal Gyrus L |  |  | 5,02 | -39 | -9 | -30 |
| Inferior Temporal Gyrus L |  |  | 4,94 | -45 | -12 | -26 |
| Superior Temporal Gyrus L | 1264 | 0,0282 | 4,26 | -60 | -35 | 23 |
| Supramarginal L |  |  | 4,01 | -60 | -36 | 33 |
| Postcentral Gyrus L |  |  | 3,92 | -36 | -29 | 44 |
| Middle Cingulate R | 1139 | 0,0326 | 3,85 | 3 | -33 | 48 |
| Supramarginal R |  |  | 3,81 | 48 | -33 | 38 |
| Postcentral Gyrus R |  |  | 3,67 | 39 | -24 | 36 |
| **AD Atrophy** | | | | | | |
| Hippocampus L | 110129 | 0,0002 | 8,99 | -21 | -5 | -20 |
| Hippocampus R |  |  | 8,37 | 20 | -5 | -18 |
| Hippocampus R |  |  | 8,34 | 24 | -8 | -18 |
| Precuneus R | 3920 | 0,005 | 4,98 | 6 | -51 | 14 |
| Middle Cingulate R |  |  | 4,36 | 0 | -35 | 39 |
| Precuneus R |  |  | 3,82 | 2 | -65 | 35 |

**Table S1.2. Gray matter atrophy patterns in patients**. Related to Fig. 1. Atrophy in each patient group was calculated via voxel-based morphometry, based on *w*-score maps of the normalized and smoothed DARTEL outputs (Chung et al., 2017; Jack et al., 1997; La Joie et al., 2012; Ossenkoppele et al., 2015; van Loenhoud et al., 2017). We ran two sample t-tests between patients and HCs using the statistical non-parametric mapping (SnPM13, http://www.fil.ion.ucl.ac.uk/spm/snpm) toolbox for SPM12, based on 5000 random permutations. Significance was set to the voxel level *P <* 0.001 for the cluster-forming threshold, and *P <* 0.05 with family-wise error correction for the cluster-wise threshold (Kim et al., 2020; Salamone et al., 2021; Shih et al., 2019). BvFTD patients showed atrophy in the right inferior and middle orbitofrontal gyri, the right insula, the right inferior and middle temporal gyri, the right middle cingulate, right supramarginal, right postcentral gyrus, left inferior and superior temporal gyri and left supramarginal. AD patients showed an extended atrophy including the bilateral hippocampus, the right precuneus, the right middle cingulate. No atrophy was found in PD patients. Results are presented on MNI space using the AAL atlas (Tzourio-Mazoyer et al., 2002), in the neurological convention. Results are plotted in **Fig. 1A.** AD: Alzheimer’s disease, bvFTD: behavioral-variant frontotemporal dementia, FWE: family-wise error, L: left, R: right.

# 2. Material S2. Task design

## 2.1. Material S2.1. Behavioral task validation

The novel domain-specific WM task (**Fig. 1B**) was designed based on previous WM paradigms (Fiebach et al., 2006; Parra et al., 2010; Pietto et al., 2016; Reuter-Lorenz et al., 2000) and programmed using Psychtoolbox-3 (Kleiner et al., 2007) in MATLAB. Stimuli were first selected: Spanish adjectives classified as social (used to describe a person when interacting with another, for example: *cordial, friendly, prudent*) and non-social (by no means used to describe a person during a social interaction, for example: *oval, rocky, printed*). All adjectives were written in male to avoid gender biases. Secondly, they were organized into online surveys distributed through Argentina, Colombia and Uruguay -Spanish speaking countries. In these surveys, 50 healthy volunteers evaluated the *sociability* of each of the words in a scale from 0 (“to no extent”) to 100 (“totally”). Social adjectives with scores equal or above 70, and non-social adjectives with scores equal or below 30 were selected. Thirdly, the chosen words were introduced into the EsPal (Spanish Lexical Database) (Duchon et al., 2013) to obtain three lexical parameters: *log frequency*, *number of letters* and *number of syllables*. These lexical parameters and the sociability scores were used to paired words and create each trial, completing 10 trials for each condition (Social triplets, non-social triplets, social quartets, non-social quartets, social quintets, and non-social quintets). Then, through one-way ANOVAs, we controlled that: social triplets, quartets and quintets were statistically paired for lexical parameters; non-social triplets, quartets and quintets were statistically paired for lexical parameters; and social and non-social stimuli of every load level were statistically paired for lexical parameters, but significantly different in sociability (see **Tables S2.1.1** and **S2.1.2**). Once the *encoding phase* stimuli were created, we calculated the Levenshtein distance between words inside each trial. This parameter is defined as the minimum number of changes required to convert one word into the other (by inserting, deleting or replacing a character in word one). We used the *enchant.utils.levenshtein* function from enchant Python package. Then we calculated the mean Levenshtein distance between words of each trial. Through a one-way ANOVA, we controlled that social and non-social trials were statistically paired for the mean Levenshtein distance (see **Table S2.1.2**).

Finally, words from the *encoding phase* were randomly shuffled (inside each trial) in order to create the *testing phase* of the task. Half of the encoding trials were randomly selected and maintained the same words, and the other half had one of the words replaced by another with the same initial syllable or letter. In this last set trials, we controlled that the Levenshtein distance between the old word and the new word for social and non-social trials were statistically paired (see **Table S2.1.3**).

## 2.1. Table S2.1.1. Stimuli lexical validation

|  | **Load level** | | | **Statistical results** | **Post-hoc comparisons** |
| --- | --- | --- | --- | --- | --- |
|  | **triplets** | **quartets** | **quintets** |  |  |
| Social stimuli | | | | | |
| ***Log frequency*** | 0.71  (0.06) | 0.61  (0.19) | 0.67  (0.10) | *F* _2,27_ = 1.34,  *P =* 0.277 | 3-5 words: *P =* 0.79  3-4 words: *P =* 0.25  4-5 words: *P =* 0.59 |
| ***Number of letters*** | 8.55  (1.23) | 8.45  (0.66) | 8.5  (0.74) | *F* _2,27_ = 0.03,  *P =* 0.971 | 3-5 words: *P =* 0.99  3-4 words: *P =* 0.96  4-5 words: *P =* 0.99 |
| ***Number of syllables*** | 3.65  (0.45) | 3.62  (0.44) | 3.7  (0.27) | *F* _2,27_ = 0.09,  *P =* 0.912 | 3-5 words: *P =* 0.95  3-4 words: *P =* 0.98  4-5 words: *P =* 0.90 |
| Non-social stimuli | | | | | |
| ***Log frequency*** | 0.69  (0.04) | 0.58  (0.13) | 0.61  (0.26) | *F* _2,27_ = 1.20,  *P =* 0.316 | 3-5 words: *P =* 0.52  3-4 words: *P =* 0.30  4-5 words: *P =* 0.91 |
| ***Number of letters*** | 7.77  (0.67) | 8.12  (0.80) | 8.42  (0.73) | *F* _2,27_ = 1.97,  *P =* 0.158 | 3-5 words: *P =* 0.13  3-4 words: *P =* 0.52  4-5 words: *P =* 0.64 |
| ***Number of syllables*** | 3.53  (0.28) | 3.62  (0.41) | 3.72  (0.32) | *F* _2,27_ = 0.75,  *P =* 0.482 | 3-5 words: *P =* 0.44  3-4 words: *P =* 0.82  4-5 words: *P =* 0.80 |

**Table S2.1.1. Stimuli lexical validation**. Related to Fig. 1. Results are presented as mean (*SD*). Between load level (triplets, quartets and quintets) comparisons on lexical parameters were assessed for each stimulus type (social and non-social) separately through one-way ANOVAs and Tukey post-hoc comparisons. Significance was set to alpha level of *P <* 0.05. 3: triplets, 4: quartets, 5: quintets.

## 2.2. Table S2.1.2. Stimuli validation per type

|  | **Stimulus type** | | **Statistical results** | **Post-hoc comparisons** |
| --- | --- | --- | --- | --- |
|  | **Social** | **Non-social** |  |  |
| ***Log frequency*** | 0.66 (0.13) | 0.63 (0.17) | F_1,58_ = 0.89,  *P =* 0.349 | Social - non-social:  *P =* 0.349 |
| ***Number of letters*** | 8.5 (0.88) | 8.1 (0.76) | F_1,58_ = 3.46,  *P =* 0.067 | Social - non-social:  *P =* 0.067 |
| ***Number of syllables*** | 3.66 (0.38) | 3.63 (0.34) | F_1,58_ = 0.11,  *P =* 0.731 | Social - non-social:  *P =* 0.731 |
| ***Sociability*** | 80.96 (5.00) | 10.79 (2.10) | F_1,58_ = 5014,  *P <* 0.001* | Social - non-social:  *P <* 0.001* |
| ***Levenshtein distance*** | 7.7 (1.14) | 7.43 (0.89) | F_1,58_ = 1.01,  *P =* 0.317 | Social - non-social:  *P =* 0.317 |

**Table S2.1.2. Stimuli validation per type.** Related to Fig. 1. Results are presented as mean (*SD*). Between trials’ stimulus type (social and non-social) comparisons on lexical parameters, sociability and mean Levenshtein distance between each trials’ words, were assessed separately through one-way ANOVAs and Tukey post-hoc comparisons. The asterisk (*) indicates significant differences with an alpha level of *P <* 0.05.

## 2.3. Table S2.1.3. Levenshtein distance between old/new words

|  | **Stimulus type** | | **Statistical results** | **Post-hoc comparisons** |
| --- | --- | --- | --- | --- |
|  | **Social** | **Non-social** |  |  |
| ***Levenshtein distance*** | 6.6 (1.24) | 6.07 (1.79) | F_1,28_ = 0.89,  *P =* 0.351 | Social - non-social:  *P =* 0.351 |

**Table S2.1.3. Levenshtein distance between old/new words.** Related to Fig. 1. Results are presented as mean (*SD*). Between stimulus type (social and non-social) comparisons on the Levenshtein distance between old and new words, were assessed through one-way ANOVAs and Tukey post-hoc. The asterisk (*) indicates significant differences with an alpha level of *P <* 0.05.

# 3. Material S3. EEG / fMRI acquisition and preprocessing

## 3.1. Table S3.1. EEG subsamples’ demographic data

| **Demographics** | | | | | | |
| --- | --- | --- | --- | --- | --- | --- |
|  | **HCs**  (*n =* 33) | **bvFTD**  (*n =* 19) | **AD**  (*n =* 27) | **PD**  (*n =* 12) | **Stats** | **Post-hoc comparisons** |
| Sex  (M:F) | 14:19 | 14:5 | 11:16 | 7:5 | *χ^2^* = 6.26,  *P =* 0.09 | HCs-bvFTD: *P =* 0.058;  HCs-AD: *P =* 0.999;  HCs-PD: *P =* 0.589 |
| Age | 72.76  (5.52) | 69.95  (10.61) | 74.19  (5.28) | 72.83  (4.45) | *F* = 1.49,  *P =* 0.22  *ηp^2^* = 0.04 | HCs-bvFTD: *P =* 0.47;  HCs-AD: *P =* 0.84;  HCs-PD: *P =* 0.99 |
| Education | 12.94  (3.67) | 13.89  (5.54) | 10.48  (4.93) | 13.92  (4.36) | *F* = 2.82,  *P =* 0.04*,  *ηp^2^* = 0.08 | HCs-bvFTD: *P =* 0.88;  HCs-AD: *P =* 0.17;  HCs-PD: *P =* 0.92 |

**Table S3.1. EEG subsamples’ demographic data.** Related to Fig. 2. Results are presented as mean (*SD*). The asterisk (*) indicates significant differences with an alpha level of *P <* 0.05. Demographic data were assessed through ANOVAs and Tukey post-hoc pairwise comparisons –except for sex, which was analyzed via Pearson’s chi-squared (χ^2^) test. Effects sizes were calculated through partial eta (*ηp*^2^). AD: Alzheimer’s disease, bvFTD: behavioral-variant frontotemporal dementia, HCs: healthy controls, PD: Parkinson’s disease.

## 3.2. Table S3.2. fMRI subsamples’ demographic data

| **Demographics** | | | | | | |
| --- | --- | --- | --- | --- | --- | --- |
|  | **HCs**  (*n =* 66) | **bvFTD**  (*n =* 19) | **AD**  (*n =*32) | **PD**  (*n =* 48) | **Stats** | **Post-hoc comparisons** |
| Sex  (M:F) (†) | 32:34 | 14:5 | 12:20 | 27:21 | χ^2^ = 6.92,  *P =* 0.07 | HCs-bvFTD: *P =* 0.09;  HCs-AD: *P =* 0.41;  HCs-PD: *P =* 0.52 |
| Age (†) | 70.53  (6.43) | 67.21  (11.8) | 74.69  (6.3) | 68.38  (9.06) | *F* = 5.10,  *P =* 0.002*  *ηp*^2^ = 0.08 | HCs-bvFTD: *P =* 0.38;  HCs-AD: *P =* 0.07;  HCs-PD: *P =* 0.48 |
| Education | 13.15  (4.20) | 13.26  (4.87) | 10.84  (4.48) | 11.65  (4.92) | *F* = 2.47,  *P =* 0.06,  *ηp*^2^ = 0.04 | HCs-bvFTD: *P =* 0.99;  HCs-AD: *P =* 0.09;  HCs-PD: *P =* 0.30 |

**Table S3.2. fMRI subsamples’ demographic data**. Related to Fig. 3. Results are presented as mean (*SD*). The asterisk (*) indicates significant differences with an alpha level of *P <* 0.05. The dagger (†) indicates variables with significant differences (*P <* 0.05) between neurodegenerative groups, precluding comparisons between them in our target measures. Demographic data were assessed through ANOVAs and Tukey post-hoc pairwise comparisons –except for sex, which was analyzed via Pearson’s chi-squared (χ^2^) test. Effects sizes were calculated through partial eta (*ηp^2^*). AD: Alzheimer’s disease, bvFTD: behavioral-variant frontotemporal dementia, HCs: healthy controls, PD: Parkinson’s disease.

## 3.3. Table S3.3. Specific neuroimaging parameters per scanner

|  | **Scanner** | | | | |
| --- | --- | --- | --- | --- | --- |
|  | **1** | **2** | **3** | **4** | **5** |
| Location | Buenos Aires, Argentina | Santiago, Chile | Santiago, Chile | Córdoba, Argentina | Colombia |
| Scanner | Philips Ingenia 3-T with standard head coil | Siemens Skyra 3-T with standard head coil | Siemens Skyra 3-T with standard head coil | Philips Ingenia CX  3-T | Philips Achieva 3-T with standard head coil |
| **MRI** | | | | | |
| Sequence | whole-brain T1-rapid anatomical 3D gradient echo volumes | whole-brain T1-rapid gradient-echo volumes | whole-brain T1-rapid gradient-echo volumes | whole-brain T1-weighted 3D spin-echo volumes | whole-brain T1-weighted Turbo Field echo volumes |
| Localization | parallel to the plane connecting the anterior and posterior commissures | parallel to the plane connecting the anterior and posterior commissures | parallel to the plane connecting the anterior and posterior commissures | parallel to the plane connecting the anterior and posterior commissures | parallel to the plane connecting the anterior and posterior commissures |
| Repetition time (TR) | 8300 ms | 1700 ms | 2400 ms | 6670 ms | 7000 ms |
| Echo time (TE) | 3800 ms | 2000 ms | 2000 ms | 3000 ms | 3000 ms |
| Flip angle | 8º | 8º | 8º | 8º | 8º |
| Slices | 160 | 208 | 192 | 165 | 350 |
| Matrix dimensions | 224 x 224 x 160 | 224 x 224 x 208 | 256 x 256 x 192 | 240 x 240 x 165 | 480 x 480 x 350 |
| Voxel size | 1 mm x 1 mm x 1 mm | 1 mm x 1 mm x 1 mm | 1 mm x 1 mm x 1 mm | 1 mm x 1 mm x 1 mm | 0.48 mm x 0.48 mm x 0.5 mm |
| **rsfMRI** | | | | | |
| Sequence | whole-brain functional spin echo volumes | whole-brain functional EP2D-BOLD pulse sequences | whole-brain functional EP2D-BOLD pulse sequences | whole-brain functional spin echo volumes | whole-brain functional field echo planar imaging (FE-EPI) |
| Localization | parallel to the anterior - posterior commissures | parallel to the anterior - posterior commissures | parallel to the anterior - posterior commissures | parallel to the anterior - posterior commissures | parallel to the anterior - posterior commissures |
| Acquisition sequence | ascending | intercalating pair-ascending first | intercalating pair-ascending first | ascending | ascending |
| Repetition time (TR) | 2640 ms | 2660 ms | 2660 ms | 2500 ms | 2000 ms |
| Echo time (TE) | 30 ms | 30 ms | 30 ms | 30 ms | 30 ms |
| Flip angle | 90° | 90° | 90° | 90° | 90° |
| Slices | 49 | 46 | 46 | 43 | 40 |
| Matrix dimensions | 80 x 80 x 49 | 76 x 76 x 46 | 76 x 76 x 46 | 80 x 80 x 43 | 80 x 80 x 40 |
| Voxel size in plane | 3 mm x 3 mm x 3 mm | 3 mm x 3 mm x 3 mm | 3 mm x 3 mm x 3 mm | 3 mm x 3 mm x 3 mm | 2.88 mm x 2.88 mm x 3 mm |
| Slice thickness | 3 mm | 3 mm | 3 mm | 3 mm | 3 mm |
| Sequence duration | 10 min | 10.3 min | 10.5 min | 10 min | 10 min |
| Number of volumes | 220 | 300 | 240 | 240 | 150 |

**Table S3.3. Specific neuroimaging parameters per scanner.** Related to Fig. 3.

## 3.4. Table S3.4. fMRI movement’ parameters

| **Movement’s parameters** | | | | | |
| --- | --- | --- | --- | --- | --- |
|  | **HCs** | **bvFTD** | **AD** | **PD** | **Stats** |
| Translation (mm) | 0.05 (0.02) | 0.06 (0.03) | 0.08 (0.03) | 0.09 (0.04) | *F* = 0.62,  *P =* 0.19 |
| Rotation (º) | 0.2 (0.05) | 0.3 (0.03) | 0.3 (0.02) | 0.4 (0.09) | *F* = 0.39,  *P =* 0.14 |

**Table S3.4. fMRI movement’ parameters.** Related to Fig. 3. Results are presented as mean (*SD*). Movement’s parameters were assessed between groups through ANOVA. Significance was set to alpha level of *P <* 0.05. None of the included participants showed head movements greater than 3 mm and/or rotations higher than 3º (Supekar et al., 2008). HCs: healthy controls, bvFTD: behavioral-variant frontotemporal dementia, PD: Parkinson’s disease, AD: Alzheimer’s disease.

# 4. Material S4. Behavioral data analysis

## 4.1. Table S4.1. Final number of trials per group * type * load

|  | **Df** | **Sum sq** | **Mean sq** | ***F*** | **Significance (*P*)** |
| --- | --- | --- | --- | --- | --- |
| Group | 3 | 6 | 2.007 | 0.44 | 0.72 |
| Type | 1 | 1.700e-27 | 1.747e-27 | 0.35 | 0.55 |
| Load | 2 | 3.500e-27 | 1.747e-27 | 0.35 | 0.70 |
| Group-by-Type | 3 | 1.280e-26 | 4.270e-27 | 0.87 | 0.45 |
| Group-by-Load | 6 | 2.560e-26 | 4.270e-27 | 0.87 | 0.51 |
| Type-by-Load | 2 | 3.500e-27 | 1.747e-27 | 0.35 | 0.70 |
| Group-by-Type-by-Load | 6 | 2.560e-26 | 4.270e-27 | 0.87 | 0.51 |

**Table S4.1. Final number of trials per group * type * load.** Related to Fig. 1. Number of remaining trials (after data curation) per conditions among groups were assessed through a mixed-model ANOVA. Significance was set to alpha level of *P <* 0.05. No significant differences were found in the final number of trials between groups (*F* = 0.44, *P =* 0.72), stimulus type (*F* = 0.35, *P =* 0.55) or load level (*F* = 0.35, *P =* 0.70), neither significant effect was found in the group-by-type-by-load interaction (*F* = 0.87, *P =* 0.51).

## 4.2. Table S4.2. Mean final number of trials among groups

| **Condition** | **HCs**  (*n =* 90) | **bvFTD**  (*n =* 42) | **AD**  (*n =* 54) | **PD**  (*n =* 59) |
| --- | --- | --- | --- | --- |
| Social  triplets | 6.87  (0.86) | 6.74  (0.80) | 6.80  (0.88) | 6.71  (0.93) |
| Social  quartets | 6.87  (0.86) | 6.74  (0.80) | 6.80  (0.88) | 6.71  (0.93) |
| Social  quintets | 6.87  (0.86) | 6.74  (0.80) | 6.80  (0.88) | 6.71  (0.93) |
| Non-social  triplets | 6.87  (0.86) | 6.74  (0.80) | 6.80  (0.88) | 6.71  (0.93) |
| Non-social  quartets | 6.87  (0.86) | 6.74  (0.80) | 6.80  (0.88) | 6.71  (0.93) |
| Non-social  quintets | 6.87  (0.86) | 6.74  (0.80) | 6.80  (0.88) | 6.71  (0.93) |

**Table S4.2. Mean final number of trials among groups**. Related to Fig. 1. Results are presented as mean (*SD*). Final number of trials per condition among groups. AD: Alzheimer’s disease, bvFTD: behavioral-variant frontotemporal dementia, HCs: healthy controls, PD: Parkinson’s disease.

## 4.3. Table S4.3. Shapiro-Wilk test for normality

| **Shapiro-Wilk test by group** | | | | |
| --- | --- | --- | --- | --- |
| **All groups** | **HCs** | **bvFTD** | **AD** | **PD** |
| *W =* 0.004,  *P =* 0.99 | *W =* 0.998,  *P =* 0.92 | *W =* 0.991,  *P =* 0.14 | *W =* 0.995,  *P =* 0.39 | *W =* 0.996,  *P =* 0.67 |
| **Shapiro-Wilk test by type** | | | | |
| **Social** | | | **Non-social** | |
| *W =* 0.998, *P =* 0.85 | | | *W =* 0.998, *P =* 0.80 | |

**Table S4.3. Shapiro-Wilk test for normality.** Related to Fig. 1. Shapiro-Wilk test for normality of the normalized IES by group and by stimulus type. AD: Alzheimer’s disease, bvFTD: behavioral-variant frontotemporal dementia, HCs: healthy controls, PD: Parkinson’s disease.

## 4.4. Table S4.4. Levene test for homoscedasticity

| **Levene test in all data** | |
| --- | --- |
| *W =* 1.543, *P =* 0.20 | |
| **Levene test by type** | |
| **Social** | **Non-social** |
| *W =* 1.424, *P =* 0.23 | *W =* 0.327, *P =* 0.80 |

**Table S4.4. Levene test for homoscedasticity**. Related to Fig. 1. Levene test for homoscedasticity of the normalized IES in all data and by stimulus type.

## 4.5. Table S4.5. Individual groups' IES comparison between type and load

| **Stimulus type** | | | | | |
| --- | --- | --- | --- | --- | --- |
| **Group** | **Social** | **Non-social** | **Statistical results** | | **Post-hoc comparisons** |
| **HCs** | -0.31  (0.84) | -0.44  (0.92) | *F* _1,89_ = 9.66,  *P =* 0.002*,  ηp^2^ = 0.10 | | Social- non-social:  *P =* 0.002* |
| **bvFTD** | 0.23  (0.99) | 0.35  (0.98) | *F* _1,41_ = 3.77,  *P =* 0.06,  ηp^2^ = 0.08 | | Social- non-social:  *P =* 0.06 |
| **AD** | 0.52  (0.97) | 0.53  (0.96) | *F* _1,53_ = 0.03,  *P =* 0.84,  ηp^2^ = 0.0007 | | Social- non-social:  *P =* 0.84 |
| **PD** | -0.05  (0.93) | -0.19  (0.95) | *F* _1,57_ = 7.05,  *P =* 0.01*,  ηp^2^ = 0.11 | | Social- non-social:  *P =* 0.01* |
| **Load level** | | | | | |
| **Group** | **triplets** | **quartets** | **quintets** | **Statistical results** | **Post-hoc comparisons** |
| **HCs** | -1.04  (0.76) | -0.42  (0.67) | 0.35  (0.58) | *F* _2,178_ = 295.91,  *P <* 0.001*,  *ηp*^2^ = 0.77 | 3-5 words: *P <* 0.001*  3-4 words: *P <* 0.001*  4-5 words: *P <* 0.001* |
| **bvFTD** | -0.19  (1.04) | 0.21  (0.92) | 0.85  (0.68) | *F* _2,82_ = 58.37,  *P <* 0.001*,  *ηp*^2^ = 0.59 | 3-5 words: *P <* 0.001*  3-4 words: *P <* 0.001*  4-5 words: *P <* 0.001* |
| **AD** | 0.1  (1.07) | 0.56  (0.89) | 0.92  (0.72) | *F* _2,106_ = 48.23,  *P <* 0.001*,  *ηp*^2^ = 0.22 | 3-5 words: *P <* 0.001*  3-4 words: *P <* 0.001*  4-5 words: *P <* 0.001* |
| **PD** | -0.59  (0.92) | -0.26  (0.83) | 0.5  (0.71) | *F* _2,114_ = 125.66,  *P <* 0.001*,  *ηp*^2^ = 0.69 | 3-5 words: *P <* 0.001*  3-4 words: *P <* 0.001*  4-5 words: *P <* 0.001* |

**Table S4.5. Individual groups' IES comparison between type and load**. Related to Fig. 1. Results are presented as mean (*SD*). The asterisk (*) indicates significant differences with an alpha level of *P <* 0.05. To validate our novel task, and confirm the predicted ‘social impairment’ and load effects in HCs, we analyzed their performance through a mixed model ANOVAs of the IES across stimulus type and load level (2 [type] * 3 [load]). To confirm the load effect, infer the patients’ understanding of the task and preclude performance effects to be confounded by other cognitive disruptions, we repeated the same analysis for bvFTD, AD and PD groups, separately. Between stimulus type (social and non-social) and load level (triplets, quartets and quintets) comparison on WM performance (normalized inverse efficiency score [IES]) was assessed for each group separately through mixed model ANOVAs (type III) and Tukey post-hoc comparisons. Effects sizes were calculated through partial eta (*ηp*^2^). As expected, HCs performed significantly better for non-social stimuli compared to social stimuli. Moreover, each group performance significantly decreased as load increased, validating our task, and precluding the main effects to be explained by a lack of the task understanding and/or other cognitive deficits confound. Results are plotted in **Figure S2.** AD: Alzheimer’s disease, bvFTD: behavioral-variant frontotemporal dementia, HCs: healthy controls, PD: Parkinson’s disease. 3: triplets, 4: quartets, 5: quintets.

# 5. Material S5. EEG and neuroimaging statistical analysis

## 5.1. Material S5.1. EEG: source localization analysis

sLORETA uses a particular scalp voltage distribution or the EEG cross-spectrum at the sensor level to compute the standardized current density at each predefined virtual sensors set in the cortical gray matter and the hippocampus (MNI 305, Brain Imaging Centre, Montreal Neurologic Institute [MNI]). sLORETA depends on the linear, weighted sum of the scalp electric potentials and uses a standardized version of the minimum norm current density estimation (Pascual-Marqui, 2002), overcoming deep-source estimation problems.

The electrodes layout (Biosemi 128) was registered onto the scalp MNI152 coordinates (Pascual-Marqui, 2007). Landmarks for registering the electrode locations were Nasion, Inion, left and right periauricular points. Location of landmarks and recording electrodes were expressed in millimeters, using the Cartesian coordinate system.

The EEG cross-spectrum was computed at the sensor level from the discrete Fourier transforms obtained for each EEG channel, using a 2s-length window. Then, the cross-spectrum was used to calculate the standardized current density at each of the 6239 voxels comprising the cortical gray matter of the reference brain. A signal-to-noise ratio of 1 was chosen for the regularization method used for the computation of the sLORETA transformation matrix (forward operator for the inverse solution problem). The lead field in sLORETA is calculated for a fixed set of 316 scalp electrodes from the 5% location system. In order to calculate the transformation matrix for any electrode layout, the lead field is interpolated to predefined electrode coordinates that do not have to correspond to the 5% positions (Pascual-Marqui, 2002).

The standardized current densities maps were obtained using a three-concentric-spheres head model, in a predefined source space of 6239 voxels (voxel size of 5 x 5 x 5 mm^3^) of the MNI average brain (Evans et al., 1993). A brain segmentation of 82 anatomic compartments (cortical areas) was implemented using the automated anatomical labeling (AAL) atlas (Tzourio-Mazoyer et al., 2002). Current densities maps of each participant were frequency-wise normalized. In sum, for each frequency, the spectral power computed in each voxel was divided by the mean spectral power (6239 voxels’ average). We finally obtained normalized current density maps to correlate with behavioral performance.

## 5.2. Material S5.2. Neuroimaging: functional connectivity analysis

To capture each network’s connectivity, we placed two bilateral seeds on cubic regions of interest (ROIs) with a 7x7x7 voxels size (Koslov et al., 2011) for each network on different MNI coordinates. Each pair of seeds was located on different MNI coordinates to capture each network’s connectivity: (a) the dorsal anterior cingulate cortex for the SN (Seeley et al., 2007) (MNI coordinates 10, 34, 24 and -10, 34, 24); (b) the right and left superior frontal gyri for the EN (Boord et al., 2017) (MNI coordinates 30, -2, 62 and -30, -2, 62); (c) the posterior cingulate cortex for the DMN (Uddin et al., 2009) (MNI coordinates 3,-54, 27 and -3,-54, 27); (d) the primary visual cortex for the VN (Saiote et al., 2016) (MNI coordinates 8, -92, 8 and -8, -92, 8); and (e) the primary motor cortex for the MN (Vahdat et al., 2011) (MNI coordinates 32, −30, 68 and -32, -30, 68). The weighted Symbolic Dependence Metric (wSDM) (Moguilner et al., 2018) coefficient was obtained across the whole time series. This non-linear connectivity measure is more robust than standard connectivity measures for characterizing neurodegenerative conditions (Moguilner et al., 2018). Then, we used standard masks (Shirer et al., 2012) to isolate the voxels typically involved in each resting-state network. Finally, we spatially averaged across all voxels to obtain one feature per network that was correlated with behavioral performance.

# 6. Material S6. EEG θ oscillations results

## 6.1. Table S6.1. Associations between EEG θ oscillations and WM load level

| **Regions** | ***r*** | ***P-FDR*** | **MNI coordinates** | | | **BA** |
| --- | --- | --- | --- | --- | --- | --- |
|  |  |  | **x** | **y** | **z** |  |
| **High load (quintets)** | | | | | | |
| ***All groups together*** | | | | | | |
| Superior frontal gyrus R | -0.524 | 0.044 | 15 | -15 | 70 | 6 |
| Superior frontal gyrus L | -0.525 | 0.044 | -20 | -5 | 70 | 6 |
| Middle cingulum R | -0.529 | 0.043 | 5 | -15 | 50 | 24 |
| Middle cingulum L | -0.533 | 0.043 | -5 | -20 | 50 | 24 |
| Precentral gyrus R | -0.525 | 0.044 | 15 | -20 | 70 | 6 |
| Precentral gyrus L | -0.527 | 0.043 | -15 | -15 | 70 | 6 |
| Paracentral lobule R | -0.259 | 0.043 | 5 | -25 | 70 | 6 |
| Paracentral lobule L | -0.527 | 0.043 | -5 | -15 | 70 | 6 |
| Postcentral gyrus L | -0.542 | 0.042 | -20 | -30 | 70 | 3 |
| Supplementary motor area R | -0.564 | 0.039 | 5 | -15 | 70 | 6 |
| Supplementary motor area L | -0.568 | 0.039 | -10 | -10 | 70 | 6 |
| Precuneus L | -0.523 | 0.045 | -5 | -40 | 70 | 4 |
| ***HCs*** | | | | | | |
| Superior frontal gyrus R | -0.742 | 0.025 | 25 | 30 | 55 | 8 |
| Middle frontal gyrus R | -0.762 | 0.023 | 35 | 30 | 50 | 8 |
| Precentral gyrus L | -0.708 | 0.029 | 30 | -5 | 55 | 6 |
| ***bvFTD*** | | | | | | |
| Cuneus L | 0.766 | 0.023 | -10 | -95 | 15 | 18 |
| Calcarine fissure L | 0.746 | 0.025 | -10 | -90 | 5 | 17 |
| Superior occipital gyrus L | 0.746 | 0.025 | -20 | -95 | 25 | 18 |
| Middle occipital gyrus L | 0.747 | 0.025 | -30 | -95 | 15 | 18 |
| ***AD*** | | | | | | |
| Inferior parietal lobe L | -0.745 | 0.025 | -55 | -55 | 40 | 40 |
| Supramarginal gyrus L | -0.744 | 0.025 | -55 | -50 | -30 | 40 |
| Middle occipital gyrus L | -0.742 | 0.025 | -35 | -60 | 30 | 39 |
| Angular gyrus L | -0.746 | 0.025 | -55 | -65 | 35 | 39 |
| ***PD*** | | | | | | |
| Superior frontal gyrus R | -0.809 | 0.019 | 15 | 25 | 40 | 9 |
| Superior frontal gyrus L | -0.761 | 0.023 | -15 | 25 | 40 | 9 |
| Medial superior frontal gyrus R | -0.799 | 0.020 | 10 | 25 | 45 | 8 |
| Medial superior frontal gyrus L | -0.795 | 0.020 | -5 | 25 | 40 | 8 |
| Middle frontal gyrus R | -0.800 | 0.020 | 25 | 25 | 45 | 8 |
| Inferior frontal (pars opercularis) R | -0.770 | 0.023 | 35 | 20 | 35 | 9 |
| Anterior cingulum R | -0.802 | 0.020 | 5 | 20 | 30 | 24 |
| Anterior cingulum L | -0.797 | 0.020 | 0 | 15 | 30 | 24 |
| Middle cingulum R | -0.812 | 0.019 | 10 | 20 | 40 | 32 |
| Middle cingulum L | -0.809 | 0.020 | 0 | 15 | 35 | 32 |
| Supplementary motor area R | -0.780 | 0.021 | 5 | 20 | 50 | 8 |
| Supplementary motor area L | -0.784 | 0.021 | -5 | 15 | 45 | 8 |
| **Low load (triplets)** | | | | | | |
| ***All groups together*** | | | | | | |
| Superior frontal gyrus R | -0.525 | 0.044 | 30 | 5 | 65 | 6 |
| Superior frontal gyrus L | -0.523 | 0.044 | -15 | -45 | 45 | 8 |
| Medial superior frontal gyrus R | -0.524 | 0.044 | 5 | 25 | 45 | 8 |
| Medial superior frontal gyrus L | -0.526 | 0.044 | -10 | 45 | 50 | 8 |
| Middle frontal gyrus R | -0.525 | 0.044 | 45 | 10 | 55 | 8 |
| Middle frontal gyrus L | -0.525 | 0.044 | -20 | 20 | 45 | 8 |
| Inferior frontal (pars opercularis) L | -0.523 | 0.044 | -35 | 5 | 30 | 6 |
| Anterior cingulum L | -0.524 | 0.044 | -5 | 10 | 30 | 24 |
| Middle cingulum R | -0.526 | 0.044 | 5 | 15 | 45 | 8 |
| Middle cingulum L | -0.533 | 0.043 | -5 | 15 | 40 | 6 |
| Precentral gyrus R | -0.525 | 0.044 | 40 | -10 | 65 | 6 |
| Precentral gyrus L | -0.524 | 0.044 | -25 | 10 | 45 | 6 |
| Postcentral gyrus R | -0.523 | 0.044 | 50 | -15 | 55 | 3 |
| Supplementary motor area R | -0.524 | 0.044 | 5 | -15 | 50 | 6 |
| Supplementary motor area L | -0.525 | 0.044 | -5 | -10 | 45 | 6 |
| ***HCs*** | | | | | | |
| Superior frontal gyrus R | -0.763 | 0.023 | 15 | 5 | 50 | 6 |
| Superior frontal gyrus L | -0.763 | 0.023 | -15 | 5 | 50 | 6 |
| Middle frontal gyrus R | -0.658 | 0.032 | 25 | 0 | 50 | 6 |
| Middle frontal gyrus L | -0.763 | 0.023 | -25 | -5 | 50 | 6 |
| Anterior cingulum R | -0.700 | 0.030 | 5 | 5 | 30 | 24 |
| Anterior cingulum L | -0.696 | 0.030 | -5 | 0 | 30 | 24 |
| Middle cingulum R | -0.766 | 0.023 | 5 | 0 | 45 | 32 |
| Middle cingulum L | -0.766 | 0.023 | -5 | 0 | 45 | 32 |
| Precentral gyrus R | -0.679 | 0.032 | 25 | -5 | 50 | 6 |
| Precentral gyrus L | -0.761 | 0.023 | -25 | -10 | 45 | 6 |
| Supplementary motor area R | -0.766 | 0.023 | 0 | 0 | 50 | 6 |
| Supplementary motor area L | -0.766 | 0.023 | -5 | 0 | 50 | 6 |
| ***bvFTD*** | | | | | | |
| Cuneus L | 0.640 | 0.035 | -15 | -85 | 35 | 19 |
| Calcarine fissure L | 0.644 | 0.035 | -10 | -70 | 20 | 17 |
| Lingual gyrus L | 0.645 | 0.035 | -15 | -90 | 0 | 18 |
| Superior occipital gyrus L | 0.640 | 0.035 | -25 | -85 | 35 | 19 |
| Middle occipital gyrus L | 0.640 | 0.035 | -15 | -100 | 10 | 18 |
| ***AD*** | | | | | | |
| Inferior parietal lobe L | -0.753 | 0.024 | -60 | -35 | 40 | 40 |
| Supramarginal gyrus L | -0.742 | 0.025 | -60 | -35 | 35 | 40 |
| ***PD*** | | | | | | |
| Medial orbital frontal R | -0.813 | 0.019 | 5 | 45 | 0 | 32 |
| Medial orbital frontal L | -0.821 | 0.018 | 5 | 30 | -10 | 32 |
| Anterior cingulum R | -0.833 | 0.017 | 5 | 25 | 15 | 24 |
| Anterior cingulum L | -0.850 | 0.015 | -5 | 20 | 20 | 24 |
| Middle cingulum L | -0.834 | 0.017 | -10 | 15 | 30 | 32 |
| Caudate L | -0.820 | 0.018 | 5 | 20 | -5 | - |

**Table S6.1. Associations between EEG θ oscillations and WM load level.** Related to Fig. 2. Pearson correlation tests (*P* ≤ 0.05 FDR-corrected) were performed to test the association between normalized current density maps in the EEG θ frequency band and WM performance (normalized inverse efficiency score [IES]) for the most extreme load level (quintets [high load] and triplets [low load]) conditions. Analyses were run in all groups together and individually per group (HCs, bvFTD, AD and PD). Results are plotted in **Figure S2**. These results were obtained with a demographically matched sample (see **Table S4.1**). AD: Alzheimer’s disease, BA: Brodmann area, bvFTD: behavioral-variant frontotemporal dementia, HCs: healthy controls, PD: Parkinson’s disease.

# Supplementary Figures

## Figure S1. Trial’s minimum threshold of 250 ms


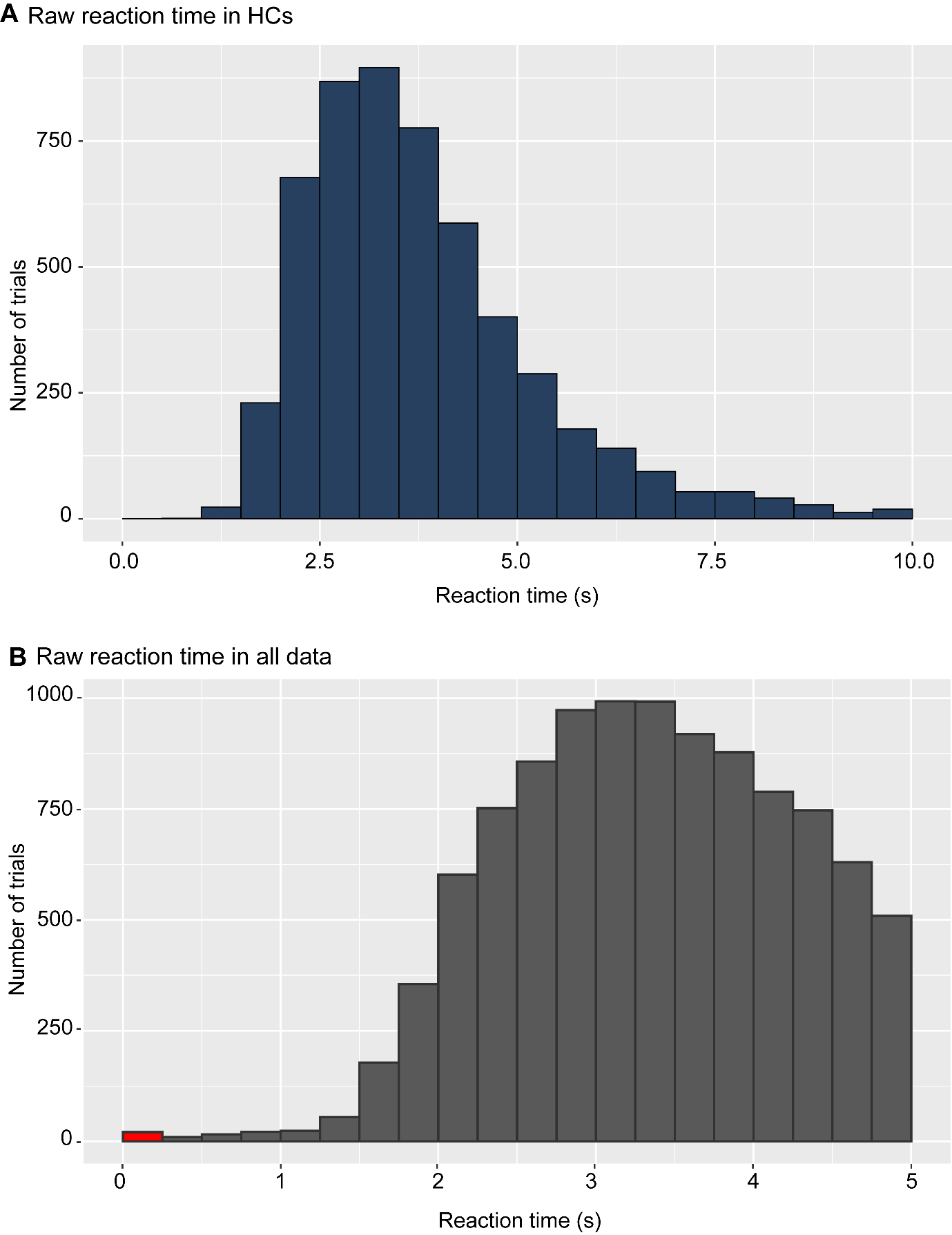


**Fig. S1. Trial’s minimum threshold of 250 ms.** Related to Fig. 1. (**A**) Raw reaction time in HCs. Considering raw trials response time in HCs (mean *=* 4.18, *SD* = 3.39, median *=* 3.54, *IQR* = 1.80), a minimum threshold of 0.250s is considered appropriate since it does not overcome the HCs median - 1.5**IQR* (0.840s). (**B**) Reaction time in raw data. Moreover, only 21 trials (colored in red) were eliminated from a total of 14940 trials, representing 0.14% of the raw data. HCs: healthy controls.

## Figure S2. Behavioral results: in-group comparisons among type and load

**
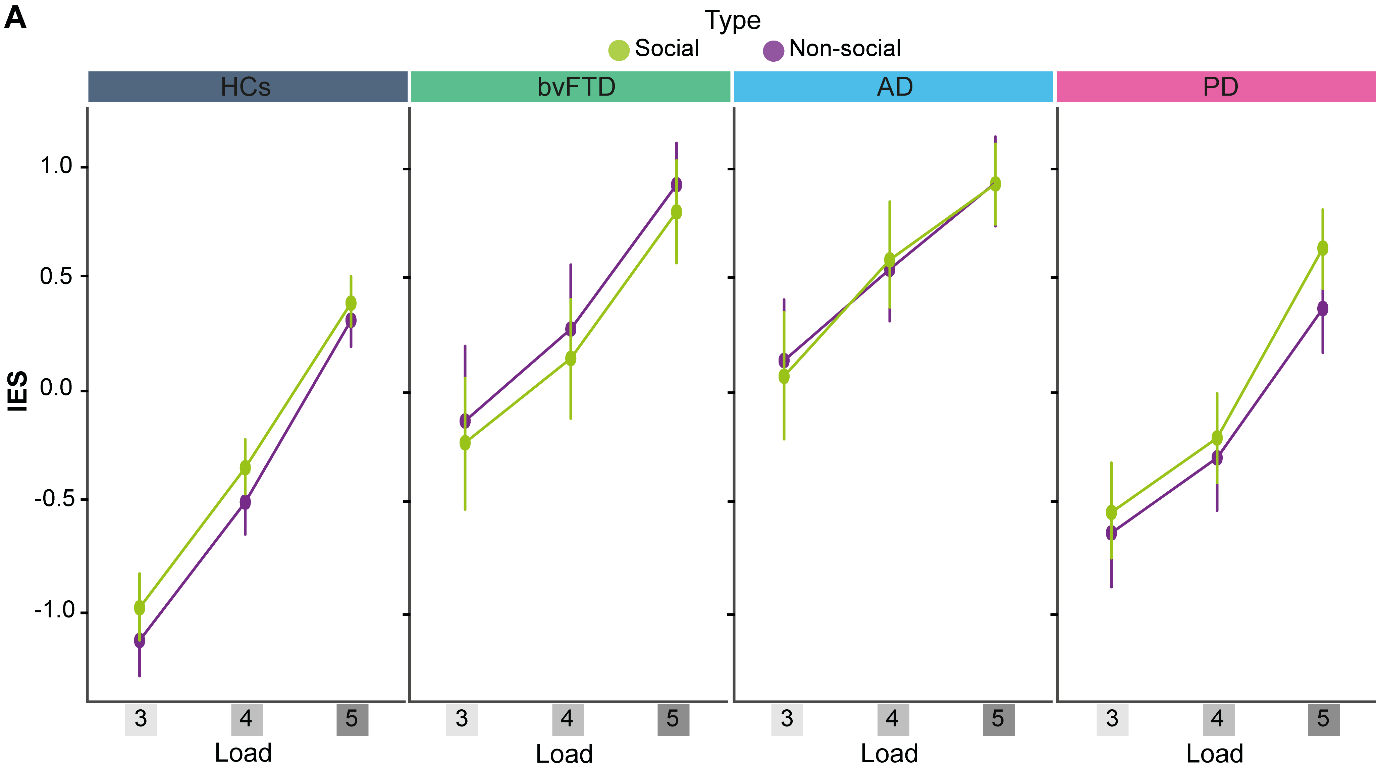
**

**Fig. S2. Behavioral results: in-group comparisons among stimulus type and load level.** Related to Fig. 1. To validate our novel task, and confirm the predicted ‘social impairment’ effect we analyzed the behavioral WM performance of HCs via a mixed model ANOVA (type [2] * load [3]) using the normalized Inverse Efficiency Score (IES). Also, to confirm the expected load effect in each group, we repeated the same analysis for bvFTD, AD and PD groups, separately. HCs performed significantly better in the non-social compared to social stimuli. Moreover, all individual groups performance significantly decreased as load increased, validating our task. For further details see **Table S4.5.** Plots represent results for HCs (dark blue), bvFTD (turquoise), AD (light blue), and PD (pink) participants. Vertical lines show mean (dot) and standard deviation (lines). AD: Alzheimer’s disease, bvFTD: behavioral variant of frontotemporal dementia, HCs: healthy controls, PD: Parkinson’s disease. 3: triplets, 4: quartets, 5: quintets. AD: Alzheimer’s disease, bvFTD: behavioral-variant frontotemporal dementia, HCs: healthy controls, IES: inverse efficiency score, PD: Parkinson’s disease.

## Figure S3. Associations between EEG θ oscillations and WM load level


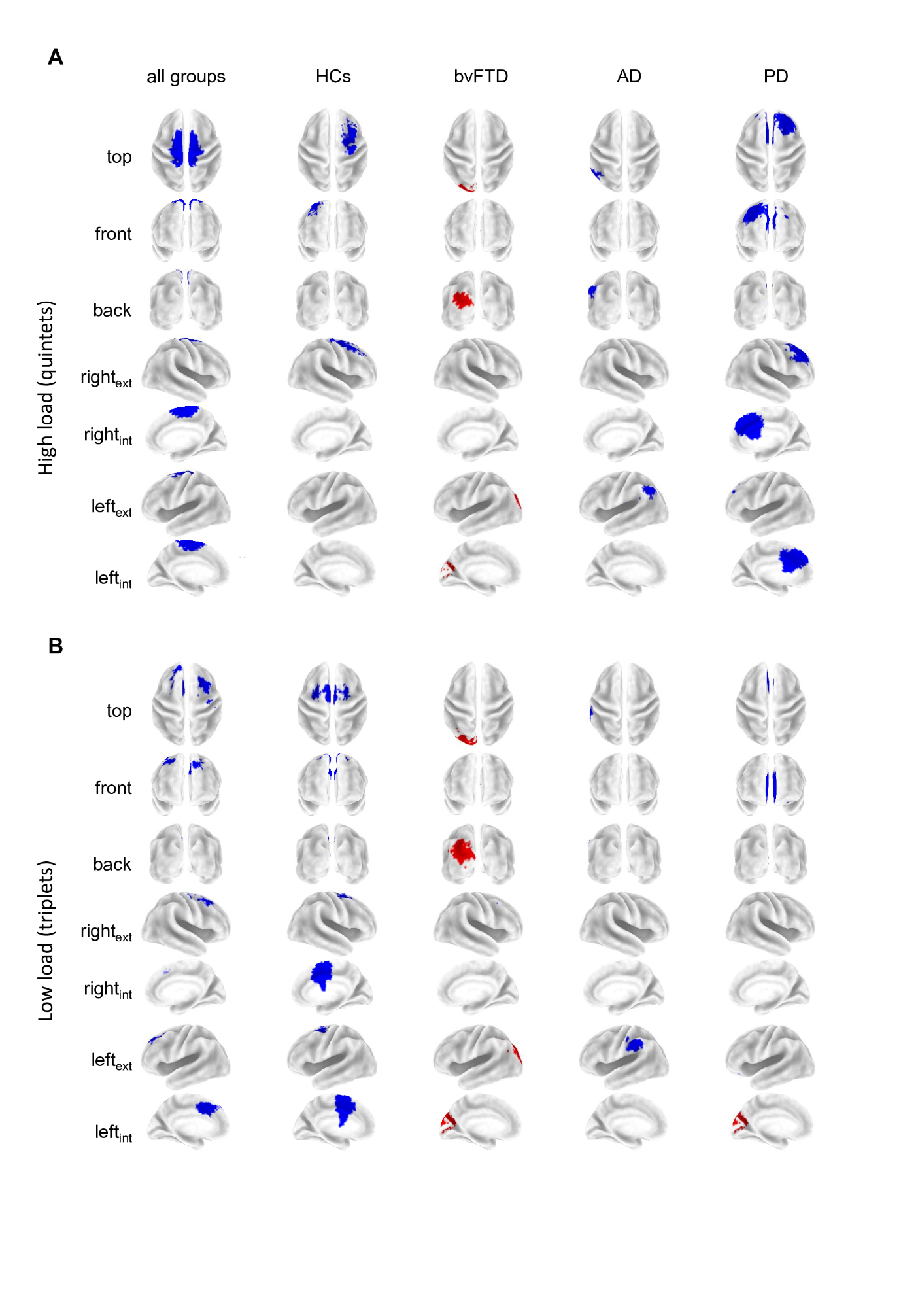


**Fig. S3.** **Associations between EEG θ oscillations and WM load level.** Related to Fig. 2. Pearson’s correlations between frequency-wise normalized current densities maps computed in the EEG θ band, and WM (normalized inverse efficiency score) for high (quintets) and low (triplets) load levels (*P* ≤ 0.05 FDR-corrected). Analyses were run in all groups together and individually per group (HCs, bvFTD, AD and PD). For further details see **Table S6.1**. Results are plotted in top, front, back, right external, right internal, left external, and left internal views. (**A**) Associations between source space EEG θ oscillations and high (quintets) load. (**B**) Associations between source space EEG θ oscillations and low (triplets) load. AD: Alzheimer’s disease, bvFTD: behavioral-variant frontotemporal dementia, HCs: healthy controls, PD: Parkinson’s disease.

## Figure S4. Associations between functional connectivity networks and WM

**
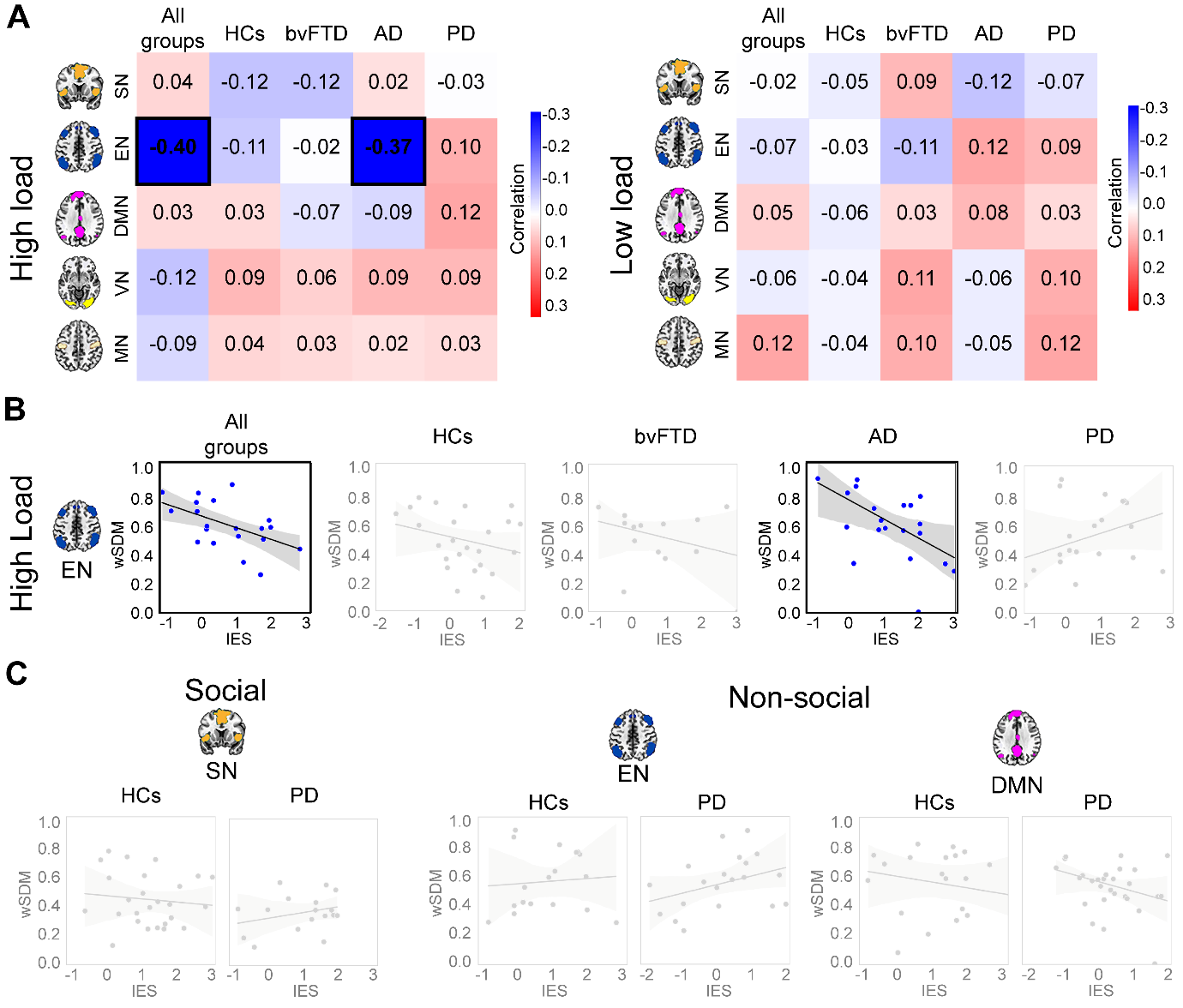
****Fig. S4. Associations between networks and WM.** Related to Fig 3. Seed analyses over five networks (SN, EN, DMN, VN, MN, *P≤*0.05 FDR-corrected) were performed to test the association between each network’ functional connectivity and WM performance (normalized IES) for social and non-social stimuli, and for high (quintets) and low (triplets) load levels. Analyses were run in all groups together and individually for HCs, bvFTD, AD and PD (**Table 5**). (**A**) **Correlation matrix for each load level.** Correlation matrix for high (quintets) and low (triplets) load levels across all groups together and individual groups. (**B**) **EN associations in high load.** Only high load (quintets) WM significantly correlated to the EN in all groups together and in AD. Null results were found across low load associations. (**C**) **Non-significant** **associations between networks and stimulus type.** Null associations were obtained between target networks (SN, EN and DMN) and WM for social and non-social stimuli in HCs and PD. For results in All groups together, bvFTD and AD see **Fig. 3**. Results were obtained with a demographically matched sample (**Table S3.2**) and across scanners (**Table S3.3**). Standard masks (Shirer et al., 2012) were used to isolate the voxels involved in each network in MNI anatomical space. None of the participants showed head movements greater than 3 mm and/or rotations higher than 3º (**Table S3.4**). AD: Alzheimer’s disease, bvFTD: behavioral-variant frontotemporal dementia, DMN: default mode network, EN: executive network, HCs: healthy controls, IES: inverse efficiency score, MN: motor network, PD: Parkinson’s disease, SN: salience network, VN: visual network, wSDM: weighted Symbolic Dependence Metric.

References

Boord, P., et al., 2017. Executive attention networks show altered relationship with default mode network in PD. NeuroImage: Clinical. 13**,** 1-8.

Chung, J., et al., 2017. Normalization of cortical thickness measurements across different T1 magnetic resonance imaging protocols by novel W-Score standardization. Neuroimage. 159**,** 224-235.

Cohen, J., 2013. Statistical power analysis for the behavioral sciences. Routledge.

Duchon, A., et al., 2013. EsPal: One-stop shopping for Spanish word properties. Behavior research methods. 45**,** 1246-1258.

Evans, A. C., et al., 3D statistical neuroanatomical models from 305 MRI volumes. 1993 IEEE conference record nuclear science symposium and medical imaging conference. IEEE, 1993, pp. 1813-1817.

Faul, F., et al., 2007. G* Power 3: A flexible statistical power analysis program for the social, behavioral, and biomedical sciences. Behavior research methods. 39**,** 175-191.

Fiebach, C. J., et al., 2006. Modulation of inferotemporal cortex activation during verbal working memory maintenance. Neuron. 51**,** 251-261.

Jack, C. R., et al., 1997. Medial temporal atrophy on MRI in normal aging and very mild Alzheimer's disease. Neurology. 49**,** 786-794.

Kim, G.-W., et al., 2020. A pilot study of brain morphometry following donepezil treatment in mild cognitive impairment: volume changes of cortical/subcortical regions and hippocampal subfields. Scientific reports. 10**,** 1-11.

Kleiner, M., et al., 2007. What's new in Psychtoolbox-3? Perception.

Koslov, K., et al., 2011. Asymmetry in resting intracortical activity as a buffer to social threat. Psychological Science. 22**,** 641-649.

La Joie, R., et al., 2012. Region-specific hierarchy between atrophy, hypometabolism, and β-amyloid (Aβ) load in Alzheimer's disease dementia. Journal of Neuroscience. 32**,** 16265-16273.

Moguilner, S., et al., 2018. Weighted Symbolic Dependence Metric (wSDM) for fMRI resting-state connectivity: A multicentric validation for frontotemporal dementia. Sci Rep. 8**,** 11181.

Ossenkoppele, R., et al., 2015. The behavioural/dysexecutive variant of Alzheimer’s disease: clinical, neuroimaging and pathological features. Brain. 138**,** 2732-2749.

Parra, M. A., et al., 2010. Visual short-term memory binding deficits in familial Alzheimer’s disease. Brain. 133**,** 2702-2713.

Pascual-Marqui, R. D., 2002. Standardized low-resolution brain electromagnetic tomography (sLORETA): technical details. Methods Find Exp Clin Pharmacol. 24**,** 5-12.

Pascual-Marqui, R. D., 2007. Discrete, 3D distributed, linear imaging methods of electric neuronal activity. Part 1: exact, zero error localization. arXiv preprint arXiv:0710.3341.

Pietto, M., et al., 2016. Behavioral and electrophysiological correlates of memory binding deficits in patients at different risk levels for Alzheimer’s disease. Journal of Alzheimer's Disease. 53**,** 1325-1340.

Reuter-Lorenz, P. A., et al., 2000. Age differences in the frontal lateralization of verbal and spatial working memory revealed by PET. Journal of cognitive neuroscience. 12**,** 174-187.

Saiote, C., et al., 2016. Resting‐state functional connectivity and motor imagery brain activation. Hum Brain Mapp. 37**,** 3847-3857.

Salamone, P. C., et al., 2021. Interoception primes emotional processing: Multimodal evidence from neurodegeneration. Journal of Neuroscience. 41**,** 4276-4292.

Seeley, W. W., et al., 2007. Divergent social functioning in behavioral variant frontotemporal dementia and Alzheimer disease: reciprocal networks and neuronal evolution. Alzheimer Disease & Associated Disorders. 21**,** S50-S57.

Shih, Y.-W., et al., 2019. Effects of positive and negative expectations on human pain perception engage separate but interrelated and dependently regulated cerebral mechanisms. Journal of Neuroscience. 39**,** 1261-1274.

Shirer, W. R., et al., 2012. Decoding subject-driven cognitive states with whole-brain connectivity patterns. Cerebral Cortex. 22**,** 158-165.

Supekar, K., et al., 2008. Network analysis of intrinsic functional brain connectivity in Alzheimer's disease. PLoS computational biology. 4.

Szucs, D., Ioannidis, J. P., 2017. Empirical assessment of published effect sizes and power in the recent cognitive neuroscience and psychology literature. PLoS biology. 15**,** e2000797.

Tzourio-Mazoyer, N., et al., 2002. Automated anatomical labeling of activations in SPM using a macroscopic anatomical parcellation of the MNI MRI single-subject brain. Neuroimage. 15**,** 273-89.

Uddin, L. Q., et al., 2009. Functional connectivity of default mode network components: correlation, anticorrelation, and causality. Hum Brain Mapp. 30**,** 625-637.

Vahdat, S., et al., 2011. Functionally specific changes in resting-state sensorimotor networks after motor learning. Journal of Neuroscience. 31**,** 16907-16915.

van Loenhoud, A. C., et al., 2017. A neuroimaging approach to capture cognitive reserve: application to Alzheimer's disease. Hum Brain Mapp. 38**,** 4703-4715.
